# EphrinB2 repression through ZEB2 mediates tumour invasion and anti-angiogenic resistance

**DOI:** 10.1038/ncomms12329

**Published:** 2016-07-29

**Authors:** C. Depner, H. zum Buttel, N. Böğürcü, A. M. Cuesta, M. R. Aburto, S. Seidel, F. Finkelmeier, F. Foss, J. Hofmann, K. Kaulich, S. Barbus, M. Segarra, G. Reifenberger, B. K. Garvalov, T. Acker, A. Acker-Palmer

**Affiliations:** 1 Institute of Neuropathology, University of Giessen, Arndtstr. 16, D-35392 Giessen, Germany; 2 Institute of Cell Biology and Neuroscience and Buchmann Institute for Molecular Life Sciences (BMLS), University of Frankfurt, Max-von-Laue-Str. 15, D-60438 Frankfurt am Main, Germany; 3 Focus Program Translational Neurosciences (FTN), University of Mainz, Langenbeckstr. 1, D-55131 Mainz, Germany; 4 Department of Neuropathology, Heinrich Heine University of Düsseldorf, Düsseldorf and German Cancer Consortium (DKTK), Moorenstr. 5, 40225 partner site Essen/Düsseldorf, Germany; 5 Division of Molecular Genetics, German Cancer Research Center (DKFZ), Im Neuenheimer Feld 580, D-69120 Heidelberg, Germany; 6 Max Planck Institute for Brain Research, Max-von-Laue Str. 4, 60438 Frankfurt, Germany

## Abstract

Diffuse invasion of the surrounding brain parenchyma is a major obstacle in the treatment of gliomas with various therapeutics, including anti-angiogenic agents. Here we identify the epi-/genetic and microenvironmental downregulation of ephrinB2 as a crucial step that promotes tumour invasion by abrogation of repulsive signals. We demonstrate that ephrinB2 is downregulated in human gliomas as a consequence of promoter hypermethylation and gene deletion. Consistently, genetic deletion of ephrinB2 in a murine high-grade glioma model increases invasion. Importantly, *ephrinB2* gene silencing is complemented by a hypoxia-induced transcriptional repression. Mechanistically, hypoxia-inducible factor (HIF)-1α induces the EMT repressor ZEB2, which directly downregulates ephrinB2 through promoter binding to enhance tumour invasiveness. This mechanism is activated following anti-angiogenic treatment of gliomas and is efficiently blocked by disrupting ZEB2 activity. Taken together, our results identify ZEB2 as an attractive therapeutic target to inhibit tumour invasion and counteract tumour resistance mechanisms induced by anti-angiogenic treatment strategies.

## Abstract (web summary)

Ephrins are transmembrane proteins involved in cell-cell communication, and implicated in cancer cell growth and progression. Here, the authors show that EphrinB2 expression is reduced in glioma cells both by genetic and epigenetic alterations and under hypoxia, through a HIF1α-mediated direct regulation of ZEB2, which enhances invasion and anti-angiogenic resistance.

## Graphical abstract

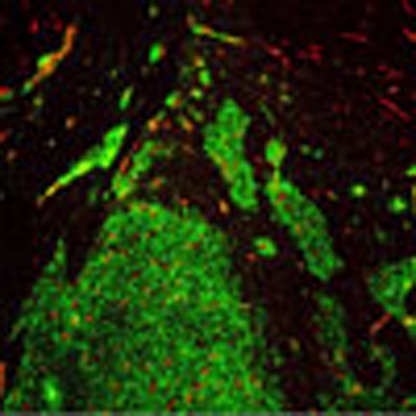

Patients suffering from high-grade gliomas, in particular, glioblastoma (GBM) multiforme, have a very poor prognosis, with a median survival of <15 months after diagnosis^1^. The aggressive behaviour of these tumours is reflected by the diffuse invasion into the surrounding brain parenchyma, which renders them incurable by operative resection and current adjuvant therapies, including radio- and chemotherapy. In the last years, anti-angiogenic therapy has emerged as a promising treatment strategy for gliomas and other solid tumours^2^,^3^. However, while initial tumour shrinkage and survival benefits in terms of time to progression are observed, these effects are transitory and tumour growth resumes^4^,^5^,^6^,^7^,^8^. Consistently, treatment of GBM patients with the angiogenesis inhibiting anti-vascular endothelial growth factor (VEGF) antibody bevacizumab is linked to enhanced invasion and multifocal tumour recurrence^7^,^8^,^9^, which is associated with aggravated mental decline. These findings urge for the identification of new targets to counteract the undesired side effects of anti-angiogenic therapy. Experimental evidence in mouse models and human patients has implicated increased invasiveness and metastasis, associated with elevated hypoxia, as an explanation for the limited efficacy of anti-angiogenic therapies^9^,^10^,^11^,^12^,^13^,^14^,^15^. Although hypoxia is a key regulator of tumour invasion by activating the transcriptional regulators hypoxia-inducible factors, HIF-1α and HIF-2α (refs 16, 17), the molecular machinery behind evasive resistance in solid tumours following anti-angiogenic therapy is insufficiently understood.

EphrinBs, transmembrane proteins that are capable of activating EphB receptors as well as transducing reverse signals, have been shown to be both up- and down-regulated in cancers^18^. Eph receptor–ephrin ligand signalling mediates repulsive effects during the separation of adjacent cell population in the development of segmented structures and in axon guidance^19^,^20^. Similarly, tumour cells need to overcome repulsive signals from the surrounding parenchyma as evidenced by the pivotal role of repulsive interactions between EphB receptors and ephrinB ligands during colon cancer progression^21^. Therefore, we hypothesized that inhibition of ephrinB function might be a crucial prerequisite to trigger tumour invasiveness. Indeed, we show that ablation of ephrinB2, increases glioma growth and invasion. We further demonstrate that loss of ephrinB2 is a common event in glioma, which can be caused by both genetic/epigenetic mechanisms and by microenvironmental triggers such as hypoxia. We identify a HIF-1α dependent mechanism that regulates ephrinB2 expression through the EMT regulator ZEB2. Importantly, silencing of ZEB2 suppresses activation of this mechanism by anti-angiogenic agents, highlighting the HIF-1α–ZEB2–ephrinB2 signalling axis as a potential target for countering tumour cell invasion and overcoming resistance against anti-angiogenic therapies.

## Results

### EphrinB2 expression is downregulated in gliomas

**Figure 1:**
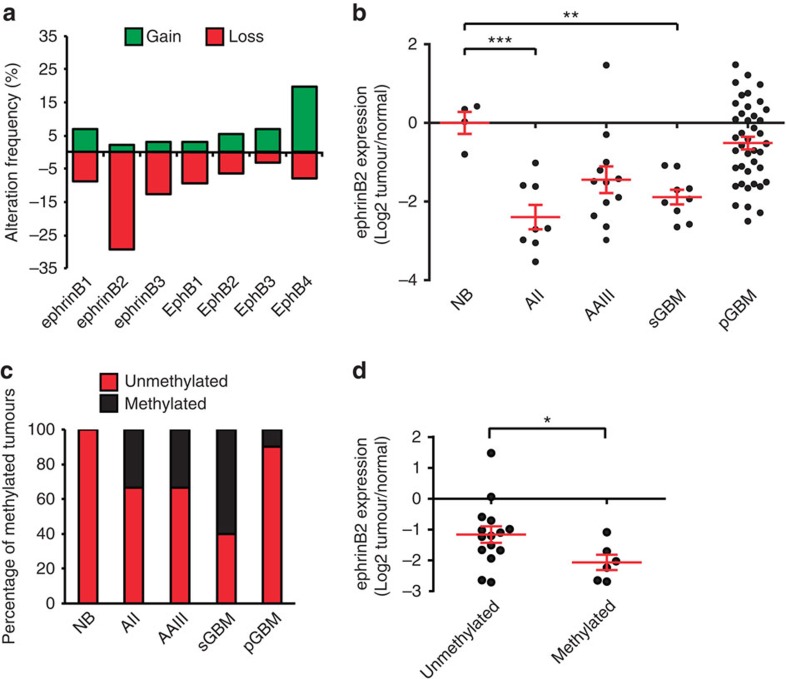
EphrinB2 expression is downregulated in gliomas. (**a**) Copy number alterations of EphB/ephrinB family members in a panel of WHO grade II, III and IV gliomas as detected by array-CGH analysis (*n*=127). (**b**) Expression of ephrinB2 mRNA relative to normal brain tissue samples in a microarray data set^22^, in a panel of WHO grade II–IV astrocytic gliomas. AII, diffuse astrocytoma, WHO grade II (*n*=8); AAIII, anaplastic astrocytoma, WHO grade III (*n*=12); NB, normal brain tissue (*n*=4); sGBM, secondary glioblastoma, WHO grade IV (*n*=9); pGBM, primary glioblastoma, WHO grade IV (*n*=41). (**c**) Frequency of ephrinB2 promoter CpG hypermethylation in normal brain and astrocytic gliomas of different WHO grades (*n*=29). (**d**) Comparison of ephrinB2 mRNA levels in gliomas with or without ephrinB2 promoter methylation (*n*=21). Data are means±s.e.m. *P<0.05, **P<0.01, ***P<0.001 using one-way analysis of variance (ANOVA) with Bonferroni post-test *P*<0.0001 (**b**, ANOVA *P*<0.0001) or Student's *t*-test (**d**).

To gain insight into the role of the EphB/ephrinB family in glioma progression, we first screened for copy number alterations of EphB/ephrinB family members by array-based comparative genomic hybridization (CGH) in a panel of 127 grade II–IV glioma patients and found the *EFNB2* gene locus to be deleted at the highest frequency (Fig. 1a). Importantly, ephrinB2 transcript levels were downregulated in gliomas relative to normal brain in a microarray data set^22^ (Fig. 1b). Alongside genetic inactivation, the epigenetic regulation by aberrant cytosine hypermethylation in CpG islands is a frequent mechanism for silencing gene expression in tumours. Sequencing of the CpG island associated with the ephrinB2 promoter region following sodium bisulfite treatment of the DNA revealed aberrant methylation of CpG sites in the tumour DNA of 34.6% of all investigated glioma patients, while no methylation was observed in normal brain (Fig. 1c). In line with an attenuation of ephrinB2 expression by promoter hypermethylation, gliomas with increased ephrinB2 CpG island methylation exhibited significantly lower ephrinB2 levels both in our glioma cohort (Fig. 1d) and in that of The Cancer Genome Atlas (TCGA, Supplementary Fig. 1a). Conversely, treatment with the DNA methyltransferase inhibitor 5-aza-2′-deoxycytidine (5-Aza-dC) and the histone deacetylase inhibitor trichostatin A significantly upregulated ephrinB2 mRNA expression in glioma cell lines, supporting the role of promoter methylation in the control of ephrinB2 expression (Supplementary Fig. 1b,c). Taken together, our results show that ephrinB2 is frequently downregulated in gliomas.

### Loss of ephrinB2 increases tumour invasiveness

**Figure 2:**
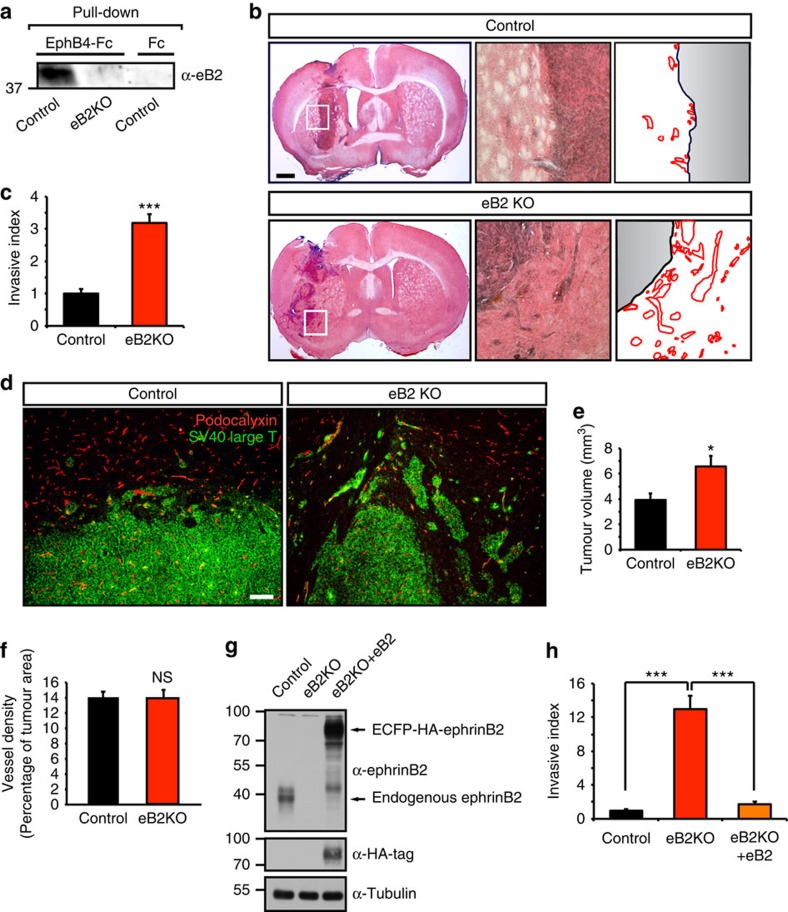
Loss of ephrinB2 increases tumour invasiveness. (**a**) Generation of gliomas lacking ephrinB2. Immunoblot of ephrinB2 knockout (eB2KO) and wild-type gliomas following pulldown of ephrinB2 by EphB4-Fc. (**b**,**c**) Loss of ephrinB2 increases tumour invasion. Hematoxylin-eosin staining of intracranial control and ephrinB2 KO glioma xenografts (left) together with representative pictures of tumour rims (middle) and traces showing central tumour tissue (grey) at the interface to the brain parenchyma (black line) and the invading cells (red lines, right) (**b**). Quantification of the invasive index of control and ephrinB2 KO gliomas, assessed by measuring the area of tumour invading the brain parenchyma per tumour rim length (*n*=5 tumours) (**c**). (**d**) Loss of ephrinB2 induces parenchymal invasion (right panel). Tumour cells are stained in green with anti-SV40 large T antigen, and endothelial cells in red with anti-podocalyxin. (**e**) Increased invasion is accompanied by increased tumour volume in ephrinB2-deficient tumours. Quantification of tumour volume in control and ephrinB2 KO gliomas (*n*=10–11 tumours). (**f**) Tumour vessel density was not affected by loss of ephrinB2 in tumour cells. Quantification of vessel area in control and ephrinB2 KO gliomas (*n*=8–10 tumours). (**g**,**h**) Re-introduction of ephrinB2 into ephrinB2 KO tumours reverts the pro-invasive phenotype. Levels of ephrinB2 assessed by immunoblot of control, ephrinB2 KO (eB2KO) and ephrinB2 KO glioma cells re-expressing exogenous ECFP-HA-ephrinB2 (eB2KO+eB2), probed with anti-ephrinB2 (**g**). Quantification of the invasive index of control, ephrinB2 KO and ephrinB2 KO gliomas re-expressing ephrinB2 was performed as in **c** (*n*=7–9 tumours) (**h**). Scale bars, 1 mm (**e**), 100 μm (**g**). Data are means+s.e.m. NS, not significant. *P<0.05, ***P<0.001 using Student's *t*-test (**c**,**e**,**f**) or one-way analysis of variance (ANOVA) with Bonferroni post-test (**h**, ANOVA *P*<0.0001).

To investigate the consequences of ephrinB2 downregulation for glioma growth, we genetically inactivated ephrinB2 in a mouse glioma model. We generated murine high-grade gliomas with a genetic deletion of ephrinB2 by immortalizing and transforming astrocytes, isolated from the cerebral hemisphere of mice homozygous for a floxed ephrinB2 allele^23^, with the SV40 large T antigen and the V12-H-ras oncogene^24^,^25^,^26^. Subsequently, ephrinB2 knockout (KO) glioma cells were obtained by adenovirus-mediated expression of Cre recombinase fused to green fluorescent protein (GFP) (Ad-Cre-GFP), whereas expression of GFP alone (Ad-GFP) yielded control ephrinB2 wild-type (WT) glioma cells. Cells were FACS-sorted and the KO was confirmed by EphB4-Fc pulldown followed by immunoblot to detect ephrinB2 (Fig. 2a). The resultant glioma cells were transplanted in an orthotopic tumour model to assess the effect of ephrinB2 deficiency on intracranial tumour growth and invasion. Notably, ephrinB2 KO tumours exhibited a markedly invasive phenotype (Fig. 2b), as confirmed by the quantification of invading tumour cells at the tumour front (Fig. 2c). EphrinB2 KO gliomas invaded as single cells, small clusters or sheets of cells protruding into the surrounding brain parenchyma (parenchymal invasion; Fig. 2d). Consistently, the expansion of tumours lacking ephrinB2 was enhanced compared with control ephrinB2 WT tumours, as evidenced by the increased tumour volume (Fig. 2e). In line with a specific pro-invasive function of ephrinB2 deficiency, tumour proliferation was not affected by ephrinB2 deletion (Supplementary Fig. 2). Importantly, tumour vascularization did not differ between ephrinB2 WT and ephrinB2 KO tumours (Fig. 2f), indicating a distinct function of ephrinB2 in tumour cells independent of its role in the tumour stroma^26^. To confirm that the pro-invasive phenotype of ephrinB2-deficient gliomas is a direct result of ephrinB2 loss of function, we performed rescue experiments by re-introducing ephrinB2 into ephrinB2 KO tumours (Fig. 2g). In line with a pivotal and direct role of ephrinB2 in the regulation of glioma invasion, ephrinB2 re-expression reversed and rescued the pro-invasive phenotype of ephrinB2 KO tumours (Fig. 2h).

We next sought to corroborate the pro-invasive effect of ephrinB2 deletion using an *ex vivo* model to assess glioma invasion. In this model glioma spheroids are seeded in brain slice cultures allowing the assessment of tumour invasion under organotypic conditions and irrespective of changes in vascularization or perfusion. In line with the pro-invasive effects observed *in vivo*, gliomas lacking ephrinB2 exhibited a drastically increased invasion of brain slices compared with control spheroids (Supplementary Fig. 3a,b). The increased propensity of ephrinB2 KO gliomas for diffuse invasion in a parenchymal environment was corroborated by co-cultivation with normal brain astrocytes expressing the ephrinB2 receptors EphB2 and EphB4 (Supplementary Fig. 4b). EphrinB2 KO glioma cells demonstrated increased dispersal and intermingling with co-cultured astrocytes and a markedly reduced tendency to remain clustered together (Supplementary Fig. 3c,d), indicating that ephrinB2 restricts the expansion of tumour cells by compartmentalization as, for example, shown for the EphB/ephrinB system in colorectal carcinoma^27^. Notably, the pro-invasive phenotype elicited by ephrinB2 deficiency was also seen in a collagen invasion assay, in which glioma spheroids lacking ephrinB2 invaded a collagen gel significantly more than WT glioma spheroids, suggesting a tumour cell intrinsic function of ephrinB2 (Supplementary Fig. 3e,f). In agreement with this, stimulation of ephrinB2 reverse signalling with its cognate receptor EphB4 decreased invasion in human glioma cell lines that express ephrinB2 (Supplementary Figs 4a,5). Collectively, our data suggest that ephrinB2 loss promotes tumour invasion by increasing the intrinsic invasive capacity and by permitting the invasion into the EphB2/EphB4 expressing parenchyma through avoiding repulsive interactions, thereby reducing tumour compartmentalization. These results identify ephrinB2 as a critical regulator of glioma invasion and establish an important role for ephrinB2 deficiency in promoting tumour invasiveness.

### HIF-1α controls the downregulation of ephrinB2 via ZEB2

**Figure 3:**
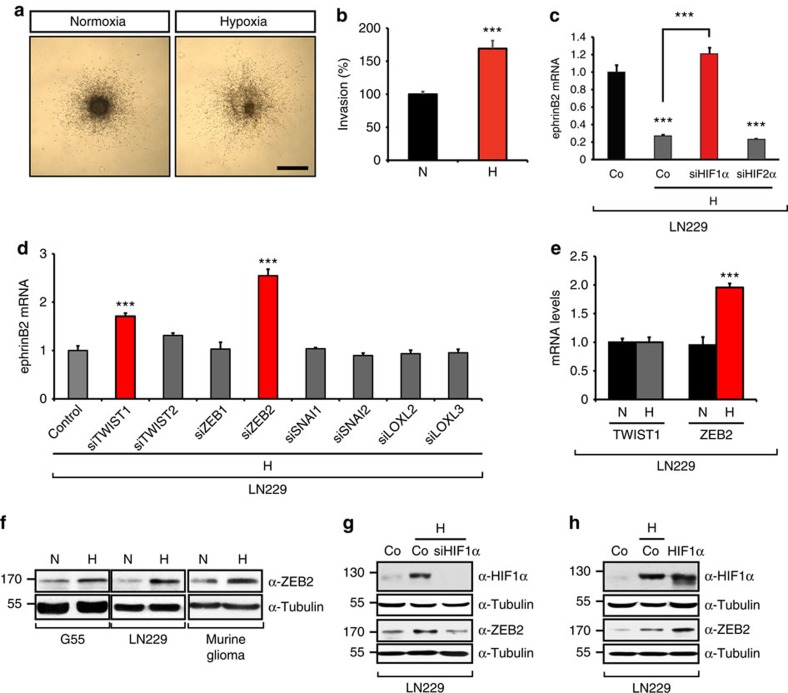
Hypoxia and HIF-1α downregulate ephrinB2. (**a**,**b**) Hypoxia induces glioblastoma invasion. Invasion of human G55 glioblastoma spheroids was quantified following incubation in a collagen gel at 21% O_2_ (N) or 1% O_2_ (H) for 48 h (*n*=7–11 spheroids). (**c**) Hypoxia downregulates ephrinB2 expression through HIF-1α. qRT–PCR analysis of ephrinB2 mRNA levels in LN229 glioblastoma cells expressing scrambled control, HIF-1α or HIF-2α shRNA following exposure to 1% O_2_ (H) for 18 h (*n*=3). (**d**) ZEB2 mediates the HIF-1α-induced downregulation of ephrinB2. siRNA-based screen of transcriptional EMT repressors in LN229 glioblastoma cells following exposure to 1% O_2_ (H) for 18 h (*n*=3); the red line marks hypoxic ephrinB2 expression levels. (**e**,**f**) Hypoxia upregulates the expression of ZEB2. qRT–PCR quantification of TWIST1 and ZEB2 mRNA levels in LN229 glioblastoma cells following exposure to 1% O_2_ (H) for 18 h (*n*=3) (**e**). Immunoblot of G55, LN229 glioblastoma and murine glioma cells at 21% O_2_ (N) or 1% O_2_ (H) probed for ZEB2 expression (**f**). (**g**,**h**) HIF-1α is required and sufficient to induce the upregulation of ZEB2. Immunoblot of LN229 glioblastoma stably transduced with scrambled control and HIF-1α shRNA (**g**) or empty vector and HIF-1α (**h**), respectively, at 21% O_2_ or 1% O_2_ (H). Western blots are representative of 2–4 independent experiments. Scale bar, 500 μm (**a**). Data are means+s.e.m. *P<0.05, **P<0.01, ***P<0.001 using Student's *t*-test (**b**,**e**) or one-way analysis of variance (ANOVA) with Bonferroni post-test (**c**,**d**, ANOVA *P*<0.0001).

Malignant tumours are characterized by regions of low oxygen (hypoxia) that lead to the activation of adaptive cellular responses including angiogenesis, glycolysis and invasion, and thereby promote tumour aggressiveness^17^. Hypoxia has also been associated with the enhanced invasion of solid tumours following anti-angiogenic treatment^13^. Indeed, exposing human glioma cells to hypoxia increased their invasive behaviour *in vitro* in collagen invasion and Boyden chamber assays (Fig. 3a,b, Supplementary Fig. 6a). We next investigated whether downregulation of ephrinB2 contributes to the highly invasive behaviour of tumours under hypoxia. Hypoxic responses are primarily regulated by the activation of the hypoxia-inducible factors HIF-1 and HIF-2 (ref. 28). Hypoxia resulted in the downregulation of ephrinB2 and this function was exclusively controlled by HIF-1α, but not HIF-2α (Fig. 3c and Supplementary Fig. 6b). Since HIF-1α is a transcriptional activator, we searched for a HIF-1α controlled transcriptional repressor that would block the expression of ephrinB2. Members of the epithelial–mesenchymal transition (EMT) regulator family are well-known for their function as transcriptional repressors and are upregulated by hypoxia and HIF^29^,^30^,^31^,^32^,^33^,^34^. Using a short interfering RNA (siRNA)-based screen against a series of transcriptional EMT repressors we determined those that mediated the hypoxia-induced downregulation of ephrinB2 (Fig. 3d). Among the EMT regulators whose inactivation abrogated the hypoxia-mediated ephrinB2 downregulation, only ZEB2 was highly upregulated by hypoxia at the mRNA and protein levels (Fig. 3e,f). Consistently, silencing of HIF-1α abrogated the hypoxic increase in ZEB2 protein levels (Fig. 3g), whereas overexpression of HIF-1α in normoxic human glioma cells was sufficient to increase the levels of ZEB2 (Fig. 3h). Taken together, our results show that hypoxia silences the expression of ephrinB2 and controls the expression of the transcriptional repressor ZEB2 through HIF-1α.

**Figure 4:**
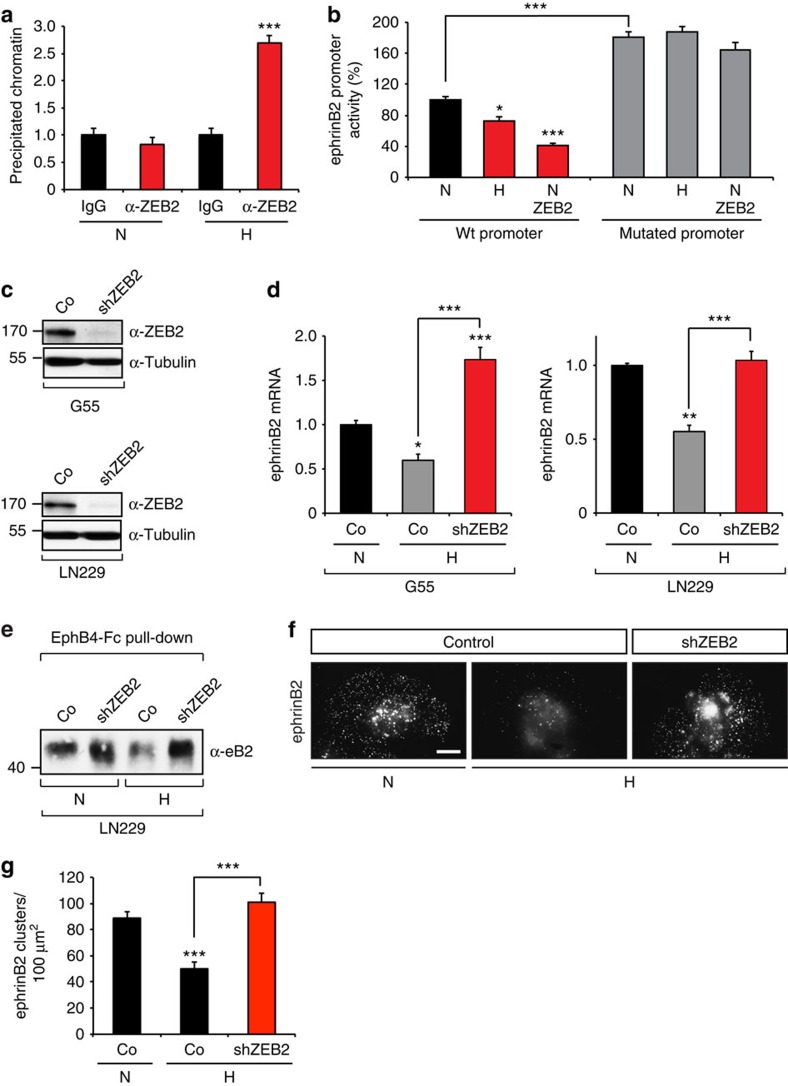
The hypoxia-induced ephrinB2 repression is mediated by ZEB2. (**a**) ZEB2 binds to the ephrinB2 promoter. qRT–PCR quantification of relative chromatin amount precipitated with anti-ZEB2 antibody compared with IgG control at 21% O_2_ (N) or 1% O_2_ (H) (*n*=5–6). (**b**) ZEB2 represses the ephrinB2 promoter activity.G55 glioblastoma cells were transfected with different murine ephrinB2 promoter constructs (containing wild-type (WT) and mutated ZEB2-binding sites), ZEB2 expression construct and exposed to 21% O_2_ (N) or 1% O_2_ (H) for 18 h. EphrinB2 promoter activity was determined by a luciferase reporter assay (*n*=3). (**c**,**d**) ZEB2 is required for the hypoxia-mediated repression of ephrinB2 mRNA levels. Immunoblot of G55 and LN229 glioblastoma cells stably transduced with scrambled control or ZEB2 shRNA, probed for ZEB2 (**c**). qRT–PCR analysis of ephrinB2 mRNA levels in G55 and LN229 glioblastoma cells expressing scrambled control or ZEB2 shRNA following exposure to 21% O_2_ (N) or 1% O_2_ (H) for 18 h (*n*=3) (**d**). (**e**–**g**) ZEB2 is required for the hypoxia-mediated downregulation of ephrinB2 protein. Immunoblot of LN229 glioblastoma cells stably transduced with scrambled control or ZEB2 shRNA at 21% O_2_ (N) or 1% O_2_ (H) for 18 h following pulldown of ephrinB2 by EphB4-Fc (**e**). EphrinB2 clusters detected by EphB4-Fc immunofluorescence in G55 glioblastoma cells stably transduced with scrambled control or ZEB2 shRNA, exposed to 21% O_2_ (N) or 1% O_2_ (H) for 18 h (**f**). Quantification representing the number of ephrinB2 clusters per 100 μm^2^ (*n*=20–30 cells) (**g**). Scale bar, 5 μm. Data are means+s.e.m. *P<0.05, **P<0.01, ***P<0.001 using Student's *t*-test (**a**) or one-way analysis of variance (ANOVA) with Bonferroni post-test (**b**,**d**,**g**; ANOVA *P*<0.0001).

To further investigate whether ZEB2 directly regulates ephrinB2 expression, we examined the promoter region of ephrinB2. We identified three conserved binding sites for ZEB2 in the promoter region of ephrinB2 (Supplementary Fig. 7a). Chromatin immunoprecipitation confirmed a specific binding of ZEB2 to the ephrinB2 promoter under hypoxic conditions (Fig. 4a, Supplementary Fig. 7b). Functionally, hypoxia and overexpression of ZEB2 in human glioma cells repressed the activity of the ephrinB2 promoter as assessed by a luciferase reporter assay (Fig. 4b). Importantly, mutation of the ZEB2-binding sites abrogated this regulation (Fig. 4b). To further corroborate the regulation of ephrinB2 expression by ZEB2 and hypoxia we silenced ZEB2 in the human glioma cell lines G55 and LN229 (Fig. 4c). ZEB2 silencing released the ephrinB2 transcriptional repression and reversed the hypoxia-induced reduction of ephrinB2 mRNA and protein levels (Fig. 4d,e). Similarly, hypoxia reduced the generation of functional membrane ephrinB2 clusters as detected by EphB4-Fc immunofluorescence, which was reversed by ZEB2 silencing (Fig. 4f,g). Collectively, our data demonstrate that hypoxia downregulates ephrinB2 expression through the HIF-1-mediated activation of the EMT transcriptional repressor ZEB2.

### ZEB2 and ephrinB2 expression at the invasive tumour front

**Figure 5:**
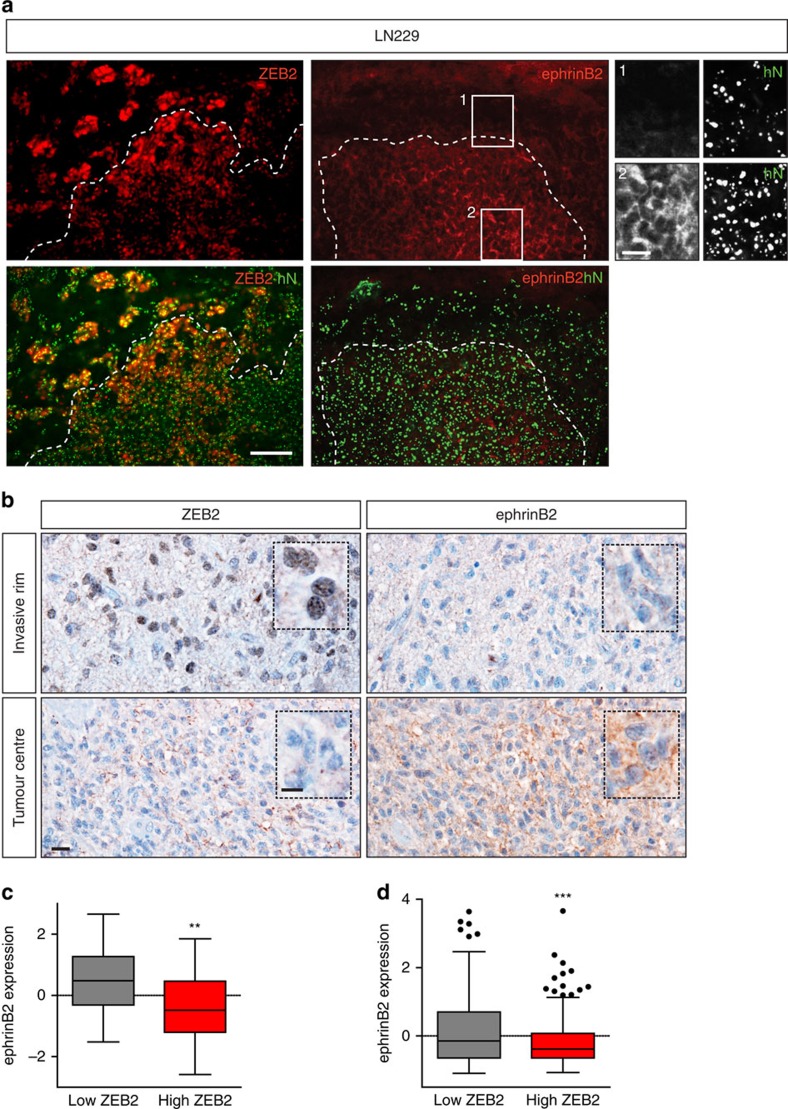
ZEB2 is upregulated and ephrinB2 downregulated at the invasive front of glioblastoma xenografts and human patients. (**a**) Immunofluorescence analysis of ZEB2 and ephrinB2 expression at the invasive tumour rim of highly invasive intracranial LN229 glioblastoma xenografts (red: anti-ZEB2, anti-ephrinB2; green: anti-human nuclei (hN) (tumour cells)). (**b**) ZEB2/ephrinB2 expression in human glioblastoma biopsies. Immunohistochemical analysis of ZEB2 and ephrinB2 expression at the invasive tumour rim and tumour centre of human glioblastoma biopsies (red-brown: ZEB2, ephrinB2; blue: nuclear counterstain hematoxylin). (**c**,**d**) The expression of ZEB2 and ephrinB2 is inversely correlated in human gliomas. EphrinB2 expression is higher in gliomas with ZEB2 expression below the median (low ZEB2) compared with gliomas with ZEB2 expression higher or equal to the median (high ZEB2), in our glioma cohort^22^ (**c**, *n*=58 gliomas, expression values are log2 tumour/normal ratios), as well as in tumors of The Cancer Genome Atlas (TCGA) cohort (**d**, *n*=530 gliomas, expression values are RNA-seq z-scores). The box-and-whiskers plots are drawn according to the method of Tukey. **P<0.01, ***P<0.001 using Student's *t*-test. Scale bars, 100 μm (**a**); 20 μm (**b** and insets in **a**).

Given that our studies identified ephrinB2 as a crucial regulator of glioma invasion, whose expression is tightly controlled by ZEB2, a member of a class of transcription factors crucially involved in the control of tumour invasion^35^, we next wanted to confirm that the ZEB2–ephrinB2 pathway is active in the regulation of glioma invasion in an *in vivo* setting. To this end, we analysed by immunofluorescence the expression of ZEB2 and ephrinB2 in the highly invasive glioma cell line LN229 following intracranial transplantation (Fig. 5a). ZEB2 was highly upregulated in glioma cells at the tumour rim and invading the adjacent brain parenchyma. Conversely, ephrinB2 was downregulated in invading tumour cells in agreement with our findings that ephrinB2 silencing promotes tumour invasion. These findings were corroborated by immunohistochemistry in human GBM biopsies (Fig. 5b). In line with the pro-invasive function of a ZEB2-mediated ephrinB2 downregulation, we observed that ZEB2 was highly upregulated in tumour cells at the invasive front, whereas ephrinB2 expression was attenuated in infiltrating glioma cells. These findings were corroborated by expression analysis in our glioma cohort and the TCGA cohort, which demonstrated an inverse correlation between the expression levels of ZEB2 and ephrinB2. Collectively, these findings implicate ZEB2 activation and ephrinB2 downregulation as crucial steps in the invasion of gliomas.

### ZEB2 directs glioma invasion and anti-angiogenic resistance

**Figure 6:**
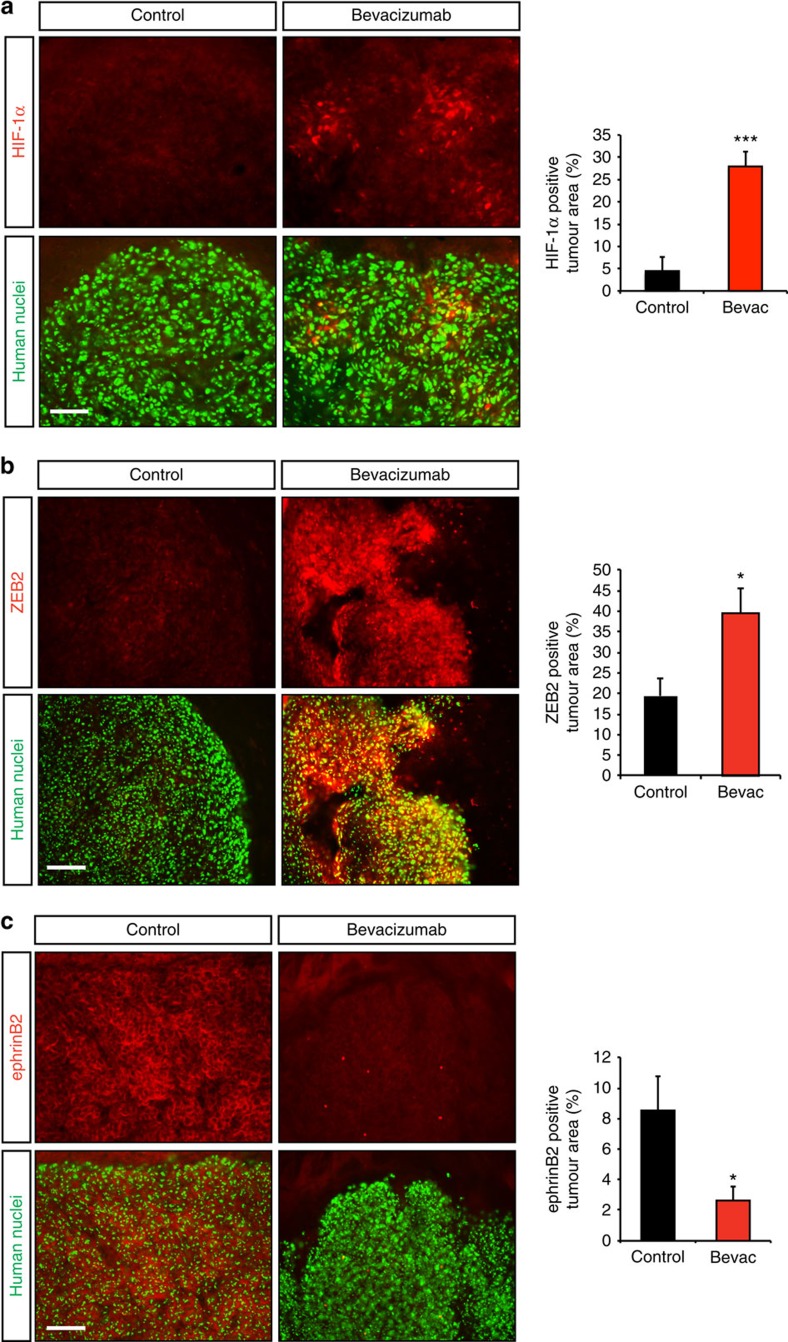
The HIF-1α/ZEB2/ephrinB2 pathway is activated following anti-angiogenic therapy. (**a**–**c**) Immunofluorescence analysis of intracranial G55 glioblastoma xenografts following IgG control or bevacizumab treatment (red: anti-HIF-1α, -ZEB2, -ephrinB2; green: anti-human nuclei (tumour cells)). The graphs represent quantifications of the tumour area positive for the respective staining (*n*=8 tumours). *P<0.05, ***P<0.001 using Student's *t*-test. Scale bars, 100 μm (**a**), 20 μm (**b**,**c**).

Previous studies have shown that VEGF-targeted anti-angiogenic therapy enhances tumour invasion, which is associated with decreased oxygen levels^9^,^10^,^13^. On the basis of our identification of the ZEB2-mediated repression of ephrinB2 as an important step in the control of tumour growth and cell invasion under hypoxia, we next explored whether anti-angiogenic treatment would activate this pathway. We used the GBM line G55 that has been previously shown to activate evasive resistance through enhanced invasion following inhibition of the VEGF pathway^36^. In line with a hypoxia-mediated increase in invasiveness following anti-angiogenic therapy, we found that HIF-1α was highly upregulated directly behind the invasive front in bevacizumab-treated gliomas (Fig. 6a). Consistently, bevacizumab treatment markedly elevated the expression of ZEB2 at the tumour rim concomitantly with increased invasiveness (Fig. 6b). Importantly, the activation of ZEB2 was accompanied by attenuation of ephrinB2 expression at the tumour rim (Fig. 6c). Taken together, these findings connect the HIF-1–ZEB2–ephrinB2 axis to the increased invasiveness observed after bevacizumab treatment.

**Figure 7:**
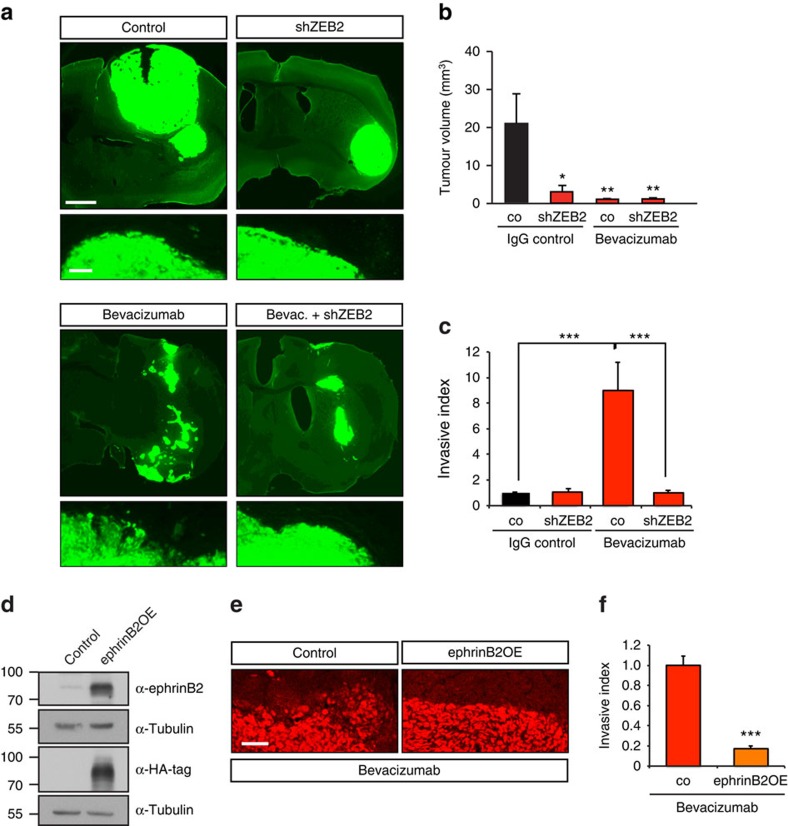
ZEB2 directs glioma invasion and anti-angiogenic resistance. (**a**–**c**) ZEB2 silencing impairs tumour growth and blocks invasion induced by anti-angiogenic treatment. Immunofluorescence analysis of intracranial tumour xenografts of GFP-expressing polyclonal G55 pools stably transduced with scrambled control or ZEB2 shRNA following IgG control or bevacizumab treatment, together with representative pictures of tumour rims (higher magnification) (**a**). Stereological quantifications of tumour volume (*n*=8 tumours) (**b**). Quantification of the invasive index assessed by measuring tumour area invading the brain parenchyma per tumour rim length (*n*=5–6 tumours) (**c**). (**d**) Immunoblot for ephrinB2 in G55 cells expressing control vector or ephrinB2 (ephrinB2OE). (**e**,**f**) EphrinB2 overexpression impairs the invasive phenotype elicited by bevacizumab. Invasive fronts of intracranial tumour xenografts of polyclonal G55 pools stably over-expressing control vector or ephrinB2 (ephrinB2OE) following bevacizumab treatment visualized using human nuclear staining (**e**). Quantification of the invasive index assessed by measuring the area of tumour invading the brain parenchyma per tumour rim length (*n*=5–9 tumours) (**f**). Scale bars, 1 mm (**a**, upper panels); 100 μm (**a**, lower panels; **e**). Data are means±s.e.m. *P<0.05, **P<0.01, ***P<0.001 using Student's *t*-test (**f**) or one-way analysis of variance (ANOVA) with Bonferroni post-test (ANOVA *P*=0.0040 (**b**) and *P*=0.0001 (**c**)).

We next addressed whether inhibiting this pathway by blocking ZEB2 would impede tumour growth and invasiveness in the context of anti-angiogenic therapy. Human G55 GBM cells with silenced ZEB2 expression were orthotopically injected in the brain of nude mice. Tumour growth was severely reduced (by 83.6%) when ZEB2 was depleted (Fig. 7a,b). Notably, ZEB2 silenced tumours appeared highly encapsulated with smooth tumour rims (Fig. 7a, higher magnifications). While ZEB2 inactivation did not affect tumour vascularization, bevacizumab treatment of G55 tumours significantly reduced vessel density (by 37%, Supplementary Fig. 8) and blocked tumour growth by 95% (Fig. 7a,b). At the same time, inhibiting angiogenesis evoked a pronounced increase in invasiveness with tumour satellites spreading through the surrounding brain parenchyma (Fig. 7a,c). Importantly, inactivation of ZEB2 completely abolished the increased invasiveness induced by bevacizumab (Fig. 7a,c) and rendered the tumours encapsulated, with smooth margins (Fig. 7a). These data confirm the crucial role of ZEB2 in the regulation of tumour invasion and are in line with the involvement of the HIF-1–ZEB2–ephrinB2 axis in the increased invasiveness following bevacizumab treatment. To further validate that the ephrinB2 downregulation following ZEB2 induction functionally regulates evasive resistance to bevacizumab treatment we generated G55 tumour cells with constitutively elevated ephrinB2 levels (Fig. 7d). Notably, ephrinB2 overexpression markedly reduced the invasive phenotype evoked by bevacizumab (Fig. 7e,f), confirming the functional requirement of attenuating ephrinB2 expression to promote invasion. Taken together, our data show that the ZEB2 induction and downregulation of ephrinB2 are important prerequisites of tumour invasion in evasive resistance.

**Figure 8:**
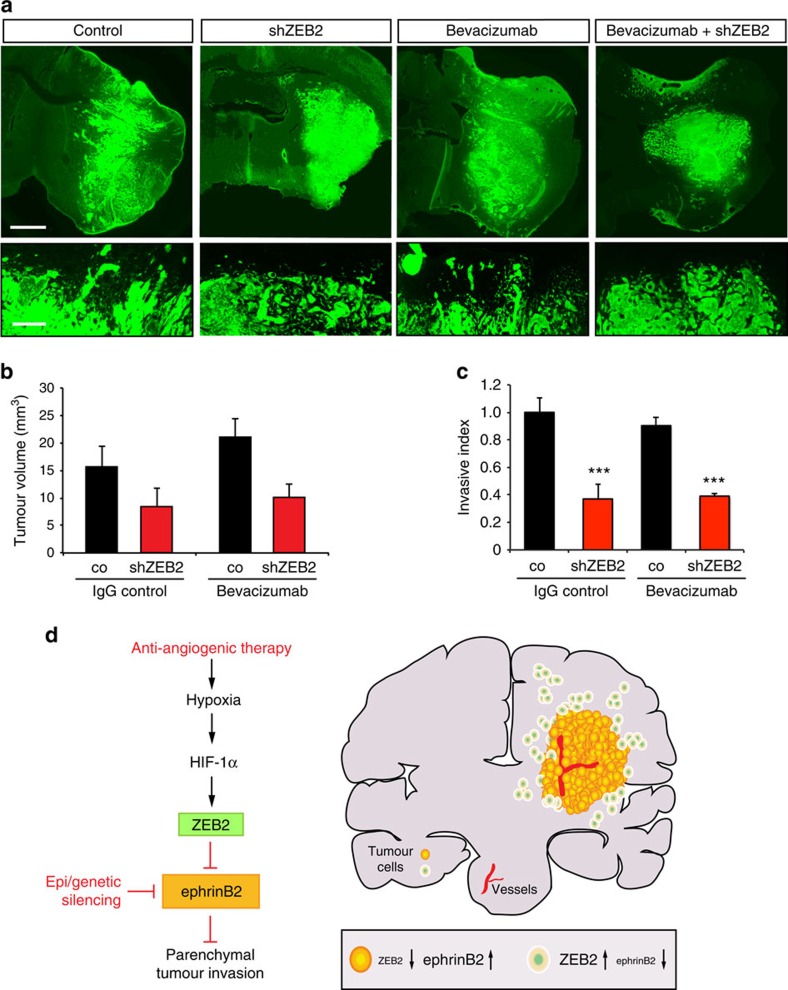
ZEB2 blockade inhibits the growth and invasion of bevacizumab-resistant LN229 glioblastoma xenografts. (**a**,**b**) ZEB2 silencing impairs tumour growth of LN229 glioblastoma xenografts. Fluorescence micrographs of intracranial tumour xenografts of GFP-expressing polyclonal LN229 pools stably transduced with scrambled control or ZEB2 shRNA following IgG control or bevacizumab treatment (**a**) and stereological quantifications of tumour volume (*n*=5–8 tumours) (**b**). (**c**) ZEB2 silencing blocks tumour invasion. Quantification of the invasive index assessed by measuring tumour area invading the brain parenchyma per tumour rim length (*n*=5–8 tumours). (**d**) Model of the regulation of glioma invasion and evasive resistance by the HIF-1α–ZEB2–ephrinB2 axis. The downregulation of ephrinB2 through genetic/epigenetic alterations and microenvironmental mechanisms is a crucial step that promotes tumour invasion by abrogation of repulsive signals. The hypoxia and HIF-1α-dependent control of ZEB2 and the ZEB2-mediated suppression of ephrinB2 is a central regulatory pathway that allows tumour cells to flexibly guide invasion in response to microenvironmental cues and promote evasive resistance to anti-angiogenic therapies in glioma. Our results identify the disruption of ZEB2 function as an attractive therapeutic strategy to inhibit tumour invasiveness and counteract anti-angiogenic resistance mechanisms. Scale bars, 1 mm (**a**), 100 μm (**a**, higher magnifications). Data are means+s.e.m. ***P<0.001 using one-way analysis of variance (ANOVA) with Bonferroni post-test; ANOVA *P*<0.0001 (**c**).

We next investigated whether ZEB2 elevation and downregulation of ephrinB2 is a general mechanism utilized in tumour invasion. We used the human glioma line LN229, which forms highly invasive tumours. Interestingly, LN229-derived tumours were naturally resistant to bevacizumab due to their lack of VEGF expression (Supplementary Fig. 9). Thus, tumour growth was not reduced and the pronounced invasiveness of untreated LN229 control tumours was not significantly altered after bevacizumab treatment (Fig. 8a,b). In contrast, ZEB2 disruption reduced tumour growth and considerably blocked tumour invasion causing the tumours to become encapsulated, with smoother rims (Fig. 8a,c), underlining the essential role of ZEB2 in tumour invasion. Collectively our findings demonstrate that the ZEB2–ephrinB2 axis is a crucial regulator of tumour invasion in glioma, whose blockade can overcome evasive resistance following anti-angiogenic treatment.

## Discussion

It is of central importance for the design of effective cancer therapies to understand the molecular mechanisms regulating tumour invasion and therapy resistance. Our work provides novel insights into these processes by revealing that the downregulation of ephrinB2 through genetic/epigenetic alterations and microenvironmental mechanisms is a crucial step that promotes tumour invasion by abrogation of repulsive signals (Fig. 8d). Importantly, we uncover an intricate link between the microenvironmental control of oxygen sensing and the repression of ephrinB2, which acts as a repulsive signal to curtail tumour cell invasion. We identify the ZEB2-mediated suppression of ephrinB2 as a central regulatory pathway in governing tumour invasion and resistance to anti-angiogenic therapies in glioma, suggesting a therapeutic target that could help counteract the development of evasive resistance.

Eph receptor–ephrin ligand signalling has been identified as a crucial pathway that controls the separation of neighbouring cell populations both during physiological development and in growing cancers, through repulsive interactions^20^,^37^. In particular, this process is well characterized in colorectal cancer progression, supporting a model in which tumour cells need to silence EphB expression to overcome the repulsive signals of ephrinB1 expressing intestinal cells to spread into the surrounding intestinal tissue^21^,^27^. Similarly to colorectal cancer, we observed that ephrinB2 is frequently silenced in GBM by gene inactivation and promoter hypermethylation, is downregulated in invading cells and that ephrinB2 silencing promotes glioma invasion. The analysis of total ephrinB2 levels in tumours is hampered by the fact that it is difficult to distinguish between the expression of ephrinB2 in the tumours cells and in tumour vessels, which are abundant in GBM and in which ephrinB2 is known to be overexpressed^38^. Therefore, we examined instead genetic and epigenetic alterations of the ephrinB2 gene that are expected to be restricted to the tumour cells, as well as localized changes in ephrinB2 expression *in situ* in invading cells compared with cells at the tumour core. Interestingly, a previous report showed using laser microdissection that ephrinB2 levels are strikingly reduced in invasive cells at the rim of human GBMs^39^. Importantly, using human glioma lines such as G55 and LN229, we demonstrate that, in addition to (epi)genetic events, hypoxia/HIF-1α represses ephrinB2 function, which is in line with the well-studied pro-invasive role of the HIF pathway and the activation of HIF-1α in invading glioma cells^17^,^40^. As shown here and in previous work^41^,^42^, ephrinB2 receptors such as EphB2 and EphB4 are expressed both in GBM cells and cells from the parenchyma, for example, astrocytes or endothelial cells. Therefore, it is likely that ephrinB2 signalling in tumour cells is influenced by the receptors in both compartments. Our findings show that loss of ephrinB2 is sufficient to induce the intrinsic invasive capacity of glioma cells. Glioma cells display complex invasive patterns with single dispersed cells, smaller or larger clusters distributed in the brain parenchyma or associated with blood vessels, and all of these modes of invasion are enhanced following loss of ephrinB2. Interestingly, ephrinB2 WT glioma cells form large cell clusters in the presence of astrocytes, whereas erphinB2 KO glioma cells grow scattered and intermingled with astrocytes, indicating that ephrinB2 restricts the expansion of tumour cells by compartmentalization, as for example, shown for the EphB/ephrinB system in colorectal cancer (see above). Our data suggest a model in which ephrinB2 loss promotes GBM invasion by increasing the intrinsic invasive capacity of tumour cells and by avoiding repulsive interactions with the EphB2/B4 expressing parenchyma, thereby reducing tumour compartmentalization.

Migration and invasion of tumours cells are tightly regulated by complex signalling networks that allow tumour cells to respond to attractive while overcoming repulsive guidance cues^18^,^32^,^43^. Members of the EMT regulating transcription factor families such as SNAI1/2, TWIST1/2 and ZEB1/2, are well-known as key inducers of invasion and metastasis in epithelial cancers through the conversion of tumour cells from an epithelial to a mesenchymal-like state. While the study of EMT has been classically confined to epithelial tumours, a rapidly growing body of recent evidence demonstrates that EMT transcription factors and an EMT-like program play a similar role in gliomas. All three main families of EMT transcription factors have been shown to be involved in the regulation of an EMT-like transition or the invasive properties of glioma cells, and various microenvironmental signals that control their activity have been identified^44^,^45^,^46^,^47^,^48^,^49^,^50^. Interestingly, there is particularly abundant evidence for the involvement of the ZEB family in glioma invasion^44^,^48^,^50^,^51^,^52^,^53^. The activation of EMT has also been linked to therapy resistance in carcinomas, and similar findings have been reported for the resistance of GBM to classical chemotherapeutics^45^,^48^. However, little is known about the possible involvement of EMT factors in the resistance to anti-angiogenic agents, such as bevacizumab, or in the anti-angiogenesis induced invasive phenotype. An important mechanism contributing to glioma invasion following VEGF inhibition was reported by Bergers and co-workers, who found that VEGF inhibits tumour cell invasion by suppressing the HGF-dependent phosphorylation of MET, which forms a complex with VEGFR2 (ref. 12). As a consequence, VEGF blockade enhances MET activity in GBM cells, which plays a critical role in inducing invasion. Importantly, the increased MET activity following VEGF ablation resulted in the acquisition of a mesenchymal phenotype, accompanied by pronounced upregulation of the transcription factor Snail. Our study now unravels a parallel crucial mechanism on how VEGF inhibition promotes glioma invasion through the induction of HIF-1α and the EMT regulator ZEB2.

Our findings support a model in which the induction of the EMT regulator ZEB2 by hypoxia and HIF-1α allows tumour cells to flexibly respond to microenvironmental cues and repress repulsive signals such as ephrinB2, enabling cells to diffusely invade the surrounding parenchyma. This is well in line with the role of the hypoxic microenvironment as a trigger for adaptive responses that endow tumours with more malignant traits. Tumour hypoxia can activate an invasive program enabling tumour cells to escape the hostile hypoxic microenvironment, as a part of an adaptive response to promote tumour survival^13^,^28^,^54^. Notably, our study highlights a function for ZEB2 in the acquisition of invasive traits, which is also activated to mediate evasive resistance to anti-angiogenic treatment in at least a subset of gliomas. Results from experimental models and clinical trials have indicated that tumours develop resistance to anti-angiogenesis that may hamper the success of this promising treatment strategy^4^,^5^,^6^,^9^,^10^,^12^,^13^,^55^,^56^. It has been established that anti-angiogenic therapies, by inhibiting blood vessel growth, induce tumour hypoxia and associated adaptive responses^9^,^57^,^58^,^59^,^60^,^61^,^62^, which can drive the development of anti-angiogenic resistance. Therefore, it has been proposed that a combination of anti-angiogenic treatment with the inhibition of the hypoxia pathway or downstream hypoxia-mediated responses can represent a crucial strategy for countering the development of anti-angiogenic resistance to improve treatment outcome^63^. Our results support the utility of such a combinatorial strategy and specifically point to disruption of ZEB2 function as an attractive therapeutic strategy to inhibit tumour invasiveness and counteract resistance mechanisms that allow tumours to evade anti-angiogenic treatment strategies.

## Methods

### Cell culture and generation of glioma lines

The cell line G55TL was kindly provided by M. Westphal and K. Lamszus (Hamburg, Germany), and the cell line LN229 by M. Weller (Zurich, Switzerland). The T98G and A172 cell lines were purchased from ATCC (N. CRL-2219, CRL-1690, CRL-1620). The human GBM cell lines were cultured with DMEM containing 10% FBS and 1% penicillin/streptomycin. Mouse glioma cells were cultured with Basal Medium Eagle (Gibco) containing 0.6% glucose, 1 mM sodium pyruvate, 10 mM HEPES, 0.5% penicillin/streptomycin, 10% horse serum (Sigma or Biochrom) and 0.1% MITO+ Serum Extender (BD). None of the cell lines used in this paper are listed in the database of commonly misidentified cell lines maintained by ICLAC. The cell lines obtained from ATCC were authenticated by the supplier.

Murine high-grade gliomas were generated by immortalization with SV40 large T antigen and transformation with H-Ras V-12, following the approach described by Blouw *et al*^24^.The generation and genotyping of conditional ephrinB2^lox/lox^ KO mice has been described^23^.Briefly, primary astrocytes were isolated from P1-2 ephrinB2^lox/lox^ mice in C57BL/6 background and plated in non-coated polystyrene culture flasks. Astrocytes were purified by shaking the flasks on a rotator at 250 rpm at 37 °C for 2 days to detach all other cells. Cells were washed and medium was changed every day. After confirmation of purity by staining with anti-GFAP antibody, astrocytes were stably co-transfected with SV40 large T antigen expression construct (pOT-largeT) and the pEYFP-N1 plasmid (Clontech) by electroporation with a nucleofector kit (Amaxa). After selection in medium containing 150 μg ml^−1^ geneticin (Gibco) for 2 weeks, resistant colonies were pooled and infected with a retrovirus expressing the H-Ras V12 oncogene (pBABEpuro H-Ras V12, Addgene). Colonies grown in selection medium containing 2 μg ml^−1^ puromycin (Sigma-Aldrich) were pooled. Expression of SV40 Large T antigen and H-Ras V12 proteins was confirmed by immunoblot analysis with corresponding antibodies (Calbiochem). The glioma cells were infected with adenovirus expressing Cre recombinase and GFP or GFP alone (Ad-Cre-GFP, Ad-CMV-GFP, Vector Biolabs) to produce ephrinB2 KO and wild-type cells. respectively. The transduced cells were purified by fluorescence activated cell sorting (FACS). EphrinB2 KO and WT gliomas were transfected with pECFP-N1 (Clontech) or pECFP ephrinB2-HA^64^ plasmids by using Fugene (Promega). Transfected cells were selected in medium containing 500 μg ml^−1^ geneticin for 2 weeks, resistant cells were pooled and ephrinB2 expression was confirmed by immunoblot analysis.

For the generation of G55 GBM cells with overexpression of ephrinB2, G55 cells were transfected with pECFP-N1 or pECFP ephrinB2-HA plasmids by using Lipofectamine 2000 (Life Technologies) and selected with 500 μg ml^−1^ geneticin for 2 weeks. EphrinB2 expression was confirmed by immunoblot analysis.

Lentiviral particles for ZEB2 shRNA were produced using pGIPZ vectors (Open Biosystems). Cells were transduced and then selected with 2 μg ml^−1^ puromycin to obtain resistant polyclonal cell pools. Lentiviral particles for HIF-1α shRNA and protein expression were produced using the pLenti6/V5-Dest vector (Block-it system, Invitrogen)^25^ and calcium phosphate transfection. Cells were transduced and afterwards selected with 6 μg ml^−1^ blasticidin to obtain resistant polyclonal cell pools. The lentiviral helper plasmids pMD2-VSV-G and pCM8.91 were kindly provided by M. Grez (Frankfurt, Germany).

### Intracranial tumour xenografts and tumour volume

All the animal experiments were conducted according to the institutional guidelines and were approved by the veterinary department of the regional council in Darmstadt Germany. Athymic female NMRI nu/nu mice at 6–8 weeks of age were anaesthetized and placed into a stereotactic apparatus (Kopf instruments). A burr hole was made 2 mm left of the sagittal suture and 0.5 mm anterior to the bregma using a dental drill with a diameter of 0.7 mm. For transplantation 5 × 10^4^ ephrinB2 WT or KO glioma cells in 1 μl, 1 × 10^5^ LN229 cells in 2 μl or 5 × 10^3^ G55TL cells in 1 μl of cold CO_2_-independent medium were slowly injected into the left striatum at a depth of 3.0 mm from the dura using a 2.5 μl Hamilton micro syringe with an unbeveled 30G needle. Transplantation experiments were performed with 8–10 mice per group. Mice transplanted with G55TL and LN229 stably transduced cells were treated with the anti-VEGF antibody bevacizumab (Avastin, Roche) or with human IgG (Gamunex 10%, Talecris Biotherapeutics) as control, respectively. Mice transplanted with stably transduced G55TL cells were injected intraperitoneally every 48 h with 20 mg kg^−1^ bevacizumab or human IgG, respectively, starting 2 days after the transplantation, mice transplanted with stably transduced LN229 cells were treated every 48–72 h with 10 mg kg^−1^ bevacizumab or human IgG, respectively, starting 1 week after the transplantation. Mice were maintained until the development of neurological symptoms prior to cardiac perfusion with 4% PFA in PBS. Tumour volume was determined by tracing the tumour area on H/E stained (glioma) or GFP positive (G55TL and LN229) slices using the semi-automated stereological system Stereo Investigator 4.34 (MicroBrightField Inc., Williston, VT, USA) or ImageJ software (NIH, http://imagej.nih.gov/ij/)on micrographs taken with Zeiss Axio Imager M1 (Carl Zeiss) and digital camera Spot Pursuit (Model 16,4 4 MP Slider, Diagnostic Instruments, Inc.). Series of every twelfth section (480 μm interval) throughout the brains were analysed.

### Analysis of copy number alterations and gene expression

The investigated glioma specimens were retrieved from the archive of the Department of Neuropathology, Heinrich Heine University, Düsseldorf, Germany, and investigated in an anonymized manner as approved by the institutional review board (study No. 4842). All tumours had been originally classified according to the criteria of the World Health Organization (WHO) classification of tumours of the nervous system of 2000, which in case of diffuse astrocytic gliomas correspond to those in the 2007 WHO classification^65^. Only tissue specimens with a histologically estimated tumour cell content of 80% or more were used for nucleic acid extraction using ultracentrifugation over caesium chloride. The analysis of ephrinB/EphB gene copy number was performed using array-based comparative genomic hybridization, and gene expression assessment was performed by microarray analysis of a panel of human glioma samples (12 diffuse astrocytomas, WHO grade II; 18 anaplastic astrocytomas, WHO grade III; 86 primary GBMs, WHO grade IV; and 11 secondary GBMs, WHO grade IV) as previously described^22^, GEO database series GSE18166; the expression data were normalized to the average expression level of 4 normal brains and expressed as log2 ratio tumour/normal tissue.

Gene expression (RNA-seq z-scores) data for the brain lower grade glioma cohort of The Cancer Genome Atlas (TCGA) were retrieved from the cBio portal^66^. Samples with an RNA-seq z-score below the median were defined as low expressing, samples with an RNA-seq z-score equal to or higher than the median—as high expressing.

### Analysis of promoter methylation

The methylation status of the *EFNB2* associated CpG island located at chromosome 13 between nucleotides 107,186,469 and 107,189,024 (http://genome.ucsc.edu) was assessed either by conventional sequencing (primary tumours) or by pyrosequencing (glioma cell lines) of sodium bisulfite-modified DNA. Sodium bisulfite treatment of 1 μg DNA was performed using the EZ DNA Methylation-Gold Kit (HISS Diagnostics, Freiburg, Germany). Two different regions of the *EFNB2* 5′-CpG island were analysed: In region 1 located between nucleotides 107,187,365 and 107,187,749 48 CpG sites were investigated for *EFNB2* methylation. In region 2 located between nucleotides 107,186,809 and 107,187,049 14 CpG sites were analysed. Aberrant methylation in tumour DNAs was mostly detected in region 2, while only individual CpG sites in region 1 showed evidence for increased methylation in the investigated tumours. Thus, calculation of the *EFNB2* promoter methylation status was based on the methylation patterns detected in region 2. The PCR fragments were amplified from sodium bisulfite-modified DNA using primers:

EFNB2-fw2 5′- gcggccgcGAYGtAGGtTGGGAtttttAATttT -3′

EFNB2-rv2 5′- CRCTaCACTaaATCTATAaTCACAaa -3′ for region 1

EFNB2-fw3 5′- gcggccgcGtttttttttAGAGtttAttAGTttt -3′

EFNB2-rv3 5′- ATTTCACTAaaTaaAaACTCCTC -3′ for region 2.

PCR products were purified using the Jetquick PCR Product Purification Kit (Genomed, Löhne, Germany). Sequencing was performed with the BigDye Cycle Sequencing Kit and an ABI PRISM 377 semi-automated DNA sequencer. To calculate the degree of CpG site methylation in the investigated DNA segment, the methylation status at each analysed CpG site was semi-quantitatively rated using the following scale: 0, completely unmethylated; 1, a weak methylated signal detectable in the sequence; 2, methylated signal approximately equal to unmethylated signal; 3, methylated signal markedly stronger than unmethylated signal. The methylation analysis was performed on 3 diffuse astrocytomas (WHO grade II), 3 anaplastic astrocytomas (WHO grade III), 10 primary GBMs (WHO grade IV) and 10 secondary GBMs (WHO grade IV), along with 3 normal brains as controls.

Glioma cell lines (A172, T98G) were grown for 24 h in Dulbecco's modified Eagle medium (DMEM, supplemented with 10% heat-inactivated fetal bovine serum and penicillin/streptomycin (Invitrogen/GIBCO, Carlsbad, CA) containing 500 nM 5-aza-2′-deoxycytidine, washed and grown again for 24 h in medium with 500 nM 5-aza-2′-deoxycytidine and 1 μM trichostatin A (A+T). Nucleic acids were purified by ultracentrifugation^67^. Pyrosequencing of sodium bisulfite-modified DNA of the cell lines A172 and T98G was performed using the PyroMark Q24 machine (Qiagen, Hilden, Germany). The corresponding oligonucleotide primers were:

EFNB2-pyro-fw 5′- GGTTAGTTTAGATTGTGGTTATG -3′

EFNB2-pyro-bio-rw 5′- CCATTTCACTAAATAAAAACTC -3′

EFNB2-pyro-seq 5′- GGAGAGGGTTAGGTA -3′.

Sequence runs as well as data analyses were performed with the PyroMark Q 24 software. The percentage of methylated alleles was calculated as the mean value of the methylation percentage obtained at each of the three investigated CpG dinucleotides located at nucleotides 107,186,901 (CpG1), 107,186,907 (CpG2) and 107,186,919 (CpG3) in the EFNB2 CpG island.

DNA methylation (HM450) data for the brain lower grade glioma cohort of The Cancer Genome Atlas (TCGA) were retrieved from the cBio portal^66^. Samples with a beta value <0.3 were defined as unmethylated, samples with a beta value >0.3 ′ as methylated.

### Analysis of protein expression

Expression of ephrinB2 protein on cells attached to coverslips was analysed by stimulation with 4 μg ml^−1^ final concentration of recombinant mouse EphB4-Fc (R&D) pre-coupled with goat anti-human IgG-Cy3 (Jackson ImmunoResearch) antibody. Micrographs of cells were taken at 100x magnification using a Zeiss Axio Imager M1 microscope. EphB4–ephrinB2 clusters were quantified with ImageJ software.

For the detection of protein expression via immunoblot analysis, cells or total brain lysates from EphB2 KO mice^68^ were lysed in LBA lysis buffer (50 mM Tris.HCl buffer, pH 7.5, 0.5% Triton X-100, 150 mM NaCl, 10 mM sodium pyrophosphate, 20 mM NaF, 1 mM sodium orthovanadate and 1% Complete protease inhibitors (Roche)) and centrifuged at 10,000 g for 10 min. Protein G Sepharose 4 Fast Flow beads (GE Healthcare) were incubated with 10 μg of antibody for 1 h at 4 °C before lysates with the same amount of total protein were added. Proteins bound to the beads were released by boiling the beads in sample buffer (200 mM Tris.HCl buffer, pH 6.8, 2% SDS (vol/vol) and 10% glycerol (vol/vol), with 2.5% β-mercaptoethanol (vol/vol). For the detection of ephrinB2, an EphB4-pull-down was performed as described^69^. Pre-clustered EphB4-Fc at a final concentration of 1 μg ml^−1^ was used to stimulate the cells for 1 h before lysis. EphB4 protein expression was detected by immunoprecipitation as described^64^. For immunoblotting, protein lysates were separated by 8 or 10% SDS-PAGE and transferred to a nitrocellulose membrane (Hybond ECL, Amersham). Analysis was performed using antibodies specific for ephrinB2 (1:2,000, R&D Systems, AF496), HIF-1α (1:1,000, BD Transduction Laboratories, 610958), HIF-2α, (1:750, Novus Biologicals, NB 100-122), ZEB2 (1:1,000, Santa Cruz Biotechnology, sc-48789 and 1:1,000, Sigma, SAB2102760), HA-tag (1:1,000, Biolegend, HA.11 Clone 16B12, 901501), EphB2 (1:500, R&D Systems, AF467), EphB4 (1:500–1:1,000, R&D Systems, AF446) actin (1:2,000, Santa Cruz, sc-1615) and tubulin (1:8,000, Dianova, DLN-09992) as a loading control. Immunoreactive bands were visualized with the ECL system (Amersham Biosciences). Uncropped scans of all western blots shown are presented in Supplementary Fig. 10 and 10 contd.

### Immunofluorescence and immunohistochemistry

To visualize blood vessels and tumour cells in a double immunofluorescence staining, free floating sections were washed in PBS and permeabilized in 0.5% Triton X/1% BSA in PBS overnight at 4 °C, free floating in a 24-well plate. Sections were then treated for 2 nights at 4 °C with anti-SV40 large T antigen antibody (1:200, Calbiochem, DP02) for tumour cell staining and podocalyxin antibody (1:100, R&D Systems, AF1556) for vessel visualization in 0.25% Triton X/1% BSA in PBS. After washing 2x in 0.01% Triton X/PBS and 1x in PBS, sections were incubated with secondary donkey anti-mouse Alexa Fluor 488 antibody (1:500, Invitrogen, A-21202) and donkey anti-goat Alexa Fluor 568 (1:500, Invitrogen, A-11057), respectively, for 4 h at room temperature, followed by a 10 min counterstain with DAPI (1:5,000, Applichem, A1001/0010) and TO-PRO-3 iodide (1:1,000, Invitrogen, T3605) and mounted in fluorescence mounting medium (Dako). The area covered by vessels was quantified with ImageJ.

To visualize hypoxic areas via HIF-1α in tumours, sections were mounted on microscope slides and dried overnight at room temperature. Sections were then rehydrated in PBS. Antigen retrieval was performed in a steamer for 5 min in citrate buffer, pH 6.0. After washing 2x in 0.01% Triton X/PBS and 1x in PBS, sections were blocked with 20% NGS/0.01% Triton X in PBS for 3 h. Sections were then treated for 2 nights at 4 °C with human nuclei antibody (1:200, Millipore/Chemicon, MAB4383) for tumour cell detection and human HIF-1α antibody (1:350, Cayman Chemical, 10006421) in 10% NGS/0.01% Triton X in PBS. After washing 2 × in 0.01% Triton X/PBS and 1 × in PBS, sections were incubated with secondary antibodies as described above. The hypoxic area and number of proliferating cells was quantified with ImageJ.

For ZEB2 and ephrinB2 staining antigen retrieval on mounted 40 μm sections was performed in a steamer with CC1 buffer (Ventana/Roche) for 30 min; for the ephrinB2 staining the sections were additionally treated with 3% H_2_O_2_ in PBS for 1 h. The sections were then permeabilized with 1% Triton X-100 in PBS, and blocked with 0.5% Triton X-100, 5% normal donkey-serum, 8% mouse-on-mouse (M.O.M.) Protein Concentrate (M.O.M. kit, Vector Laboratories) in PBS. Rabbit anti-ZEB2 antibody (1:200, Santa Cruz, sc-48789) or goat anti-ephrinB2 antibody (1:50, R&D Systems, AF496) were incubated together with a mouse anti-human nuclei monoclonal antibody (1:200, Millipore/Chemicon, MAB4383) in blocking buffer for 2 nights at 4 °C. After washing with 0.01% Triton X-100 in PBS, the slides were incubated for 4 h to overnight at 4 °C with donkey anti-mouse Alexa 488 (1:500, Invitrogen, A-21202) together with donkey anti-rabbit Alexa 568 (1:500, Invitrogen, A-11011; ZEB2 staining) or donkey anti-goat Alexa 568 (1:500, Invitrogen, A-11057, ephrinB2 staining) secondary antibodies. After nuclear staining with a mixture of DAPI and TO-PRO-3 (Invitrogen), the sections were mounted in fluorescence mounting medium (Dako). Imaging was performed on a Leica DM2500 fluorescence microscope.

Immunohistochemical staining of ZEB2 and ephrinB2 in GBM biopsies (*n*=5) was performed on 4 μm paraffin sections in a BenchMark XT automated staining platform (Ventana/Roche) using antigen retrieval with CC1 buffer (Ventana/Roche) and incubation with a rabbit anti-ZEB2 antibody (1:200, Santa Cruz, sc-48789) or goat anti-ephrinB2 antibody (1:50, R&D Systems, AF496) according to the manufacturer's instructions. In all immunohistochemical experiments omission of the primary antibody served as the negative control. Immunoreactivity was visualized using DAB (Roche). Sections were briefly counterstained in hematoxylin.

To visualize cell proliferation in tumours, mitotic tumour cells were stained with phospho-histone H3 primary antibody (1:200, # IHC-00061 Bethyl Laboratories, Inc.) in blocking buffer for 2 days at 4 °C. Cell proliferation was quantified by automated counting of phospho-histone H3 positive cells using ImageJ, respectively, in high magnification optical fields.

### Analysis of tumour invasion *in vitro* and *in vivo*

Human GBM invasion was assessed using the Transwell invasion system^70^. The Transwell filters were coated with a thin layer (40 μl) of Matrigel (0.18 μg μl^−1^) containing 0.74 μg pre-coupled EphB4-Fc by drying overnight. After 18–24 h starvation cells were seeded to 80% confluence on the filters rehydrated with 40 μl medium. The upper compartment was filled with 1% FCS, the lower with 10% FCS supplemented medium. After 8 to 15 h the filters were stained with DAPI. After imaging with a Zeiss Axio Imager M1 at 4 or × 2.5 magnification, the number of invading cells was quantified manually or automatically with ImageJ. Human GBM invasion was analysed using G55 spheroids produced by culturing cells under neurosphere conditions. Cell culture dishes and plates were coated with 10 mg ml^−1^ pHEMA, dried and rinsed with PBS. The neurosphere medium DMEM-F12, (Gibco, Invitrogen, Carlsbad, CA) was supplemented with 2% B-27 Serum-Free Supplement (Gibco), 20 ng ml^−1^ bFGF and 20 ng ml^−1^ EGF (PeproTech, Hamburg, Germany). G55 spheroids were then picked and plated in a 24-well in a collagen gel pH 7.4, consisting of 80% PureCol bovine collagen type I (INAMED, Fremont, Ca, USA), 10% MEM (serum free), 10% NaHCO_3_ buffer in 1x PBS and 10 μg ml^−1^ Fibronectin from bovine plasma (Sigma). Collagen was polymerized for 1 h at 37 °C and was then covered with DMEM containing 10% FCS. For hypoxic experiments the plates were incubated in a hypoxic chamber (Hypoxic Workstation Invivo2 500, Ruskinn Technology) at 1% O_2_ for 48 h. Images were taken at × 4 magnification at different time points as indicated. The area covered by invading cells was quantified with ImageJ and normalized to the perimeter of the initial spheroid. Mouse glioma invasion was analysed using a three dimensional spheroid collagen invasion assay. Spheroids were produced with 5,000 cells hanging in a drop of 25 μl medium for 2–3 days. They were plated in a 96-well in a collagen gel consisting of 1.53 mg ml^−1^ rat tail collagen type I, 1,8% horse serum, 136 mM HEPES in 1x DMEM. After 1–1.5 days of invasion images were taken at 4x magnification with a Leica DM IL inverted microscope. The area covered by invading cells was quantified with ImageJ and normalized to the perimeter of the initial spheroid.

For astrocyte:glioma co-culture experiments, primary isolated mouse brain astrocytes were seeded (5 × 10^4^ cells per cm^2^) on 1 μg ml^−1^ fibronectin from bovine plasma (Sigma-Aldrich Co., USA, Missouri, St Louis #F4759) coated coverslips, and incubated at 37 °C and 5% CO_2_ for 24 h. CFP-WT and CFP-eB2KO gliomas were seeded on top of the astrocytes in a 1:3 (astrocyte:glioma) ratio. The co-culture was maintained for the following 48 h in the same conditions as ab initio. Cells were then fixed for 20 min with PBS-4% PFA and astrocytes were stained for GFAP expression while gliomas were imaged by CFP expression. Images from 4–5 representative optical fields per sample were taken by using an AX 10 fluorescence microscope (Carl Zeiss AG, Germany, Göttingen). Cell quantification was done with ImageJ by counting the number of cells present in each CFP-positive cluster.

Mouse glioma invasion *ex vivo* was analysed using a glioma spheroid invasion assay in organotypic brain slice cultures. Glioma spheroids were produced by hanging-drop culture using 5,000 cells in 25 μl medium for 2–3 days. Organotypic corticostriatal slice cultures were prepared from 6–8 week old C57BL/6 mice. Briefly, mice brains were transferred to ice-cold dissection medium, consisting of minimum essential medium (MEM, Gibco) supplemented with 2 mM GlutaMAX (Gibco), 25 mM HEPES, 0.45% D-glucose, Penicillin/Streptomycin (Gibco) and the pH was adjusted to 7.4. Brains were sectioned into 300 μm thick coronal slices using a vibratome (Leica, Mannheim, Germany) mounted onto porous nitrocellulose filters (0.45 μm pore size, Millipore) and transferred to six well plates (NUNC). Each well contained 1 ml of pre-warmed culture medium, consisting of 42% MEM (Gibco), 25% basal medium eagle (BME, Gibco), 25% Horse Serum (Biochrom), supplemented with 25 mM HEPES, 2 mM GlutaMAX (Gibco), 0.68% D-glucose, Penicillin/Streptomycin (Gibco) and 1.5 g l^−1^ sodium bicarbonate. Brain slices were incubated in a standard humidified incubator supplied with 5% CO_2_ at 37 °C in culture medium. After 1 h recovery, single glioma spheroids were placed on the striatum region of the slices on each brain hemispheric side (control and ephrinB2 KO). Organotypic brain slices with implanted glioma spheroids were cultured in culture medium, which was changed every 2 days. Invasion of the spheroids was analysed after 5 days in culture. Brain slices were washed in PBS, fixed in 4% PFA and mounted in fluorescence mounting medium (Dako, Glostrup, Denmark). Samples were imaged in a Leica TCS SP5 confocal microscope. Z-stacks were obtained at × 20 magnification by acquiring single plane images every 5 μm through the tissue. To quantify the spheroids' invasiveness, fluorescence intensity in concentric areas was analysed with ImageJ. The fluorescence intensity was measured in 4 concentric areas. The innermost circular area had a radius of 0.3 mm, which corresponds to the average spheroids' size at the moment of implantation in the brain slices. The following areas had radii increments of 0.3 mm each. The fluorescence intensity in each of the radial strips was calculated by subtracting the intensity of the smaller preceding circular areas. In this way, the invasiveness was measured by means of fluorescence intensity in an area within a certain distance from the spheroid midpoint. Statistical significance was assessed using the *t*-test and 8 spheroids were analysed per condition.

Tumour invasion was quantified by tracing the tumour area invading the parenchyma on H/E stained (glioma), GFP positive or human nuclear staining (G55TL and LN229) slices using the MetaMorph software on micrographs taken with Zeiss Axio Imager M1 (Carl Zeiss) and digital camera Spot Pursuit (Model 16,4 4 MP Slider, Diagnostic Instruments, Inc.). The tumour invasive index was calculated as a function of the area of tumour invading the parenchyma per tumour rim length.

### BrdU proliferation assay

75,000 cells were split on coverslips in a 24-well plate. After starvation in medium without FCS for 20–24 h the cells were washed with DMEM containing 0.5% FCS and stimulated for 7 h with clustered EphB2-Fc at 37 °C in DMEM/0.5% FCS. 5 h after the beginning of stimulation 1.5 μl BrdU (10 mg ml^−1^) was added and incubated for 2 h. The cells were then fixed with ice-cold MeOH for 10 min, washed 3 × with PBS, permeabilized with 2 M HCl for 10 min and washed again 3 × with PBS. Blocking was done with 5% sheep serum+5% goat serum in PBS, followed by incubation with mouse anti-BrdU antibody (Roche, 11170376001, diluted 1:50 in blocking solution) for 1.5 h, three 5 min washes with PBS, incubation with goat anti-mouse-Alexa488 (1:200, Invitrogen, A-11029) and DAPI (1:2,000) for 1 h and three 5 min washes with PBS, after which the coverslips were mounted in Gel Mount mounting medium (Biomeda).

### Real time quantitative RT–PCR

RNA was extracted with the RNeasy Mini Kit (Qiagen), and reverse transcribed using standard protocols (Superscript II; Life Technologies). Complementary DNA was amplified using SYBR Green Mix (ABgene). Gene specific PCR products were measured with an iCycler iQ (Bio-Rad), LightCycler (Roche) or OneStepPlus (Applied Biosystems) systems. The relative mRNA level of the gene of interest was normalized to HPRT or GAPDH mRNA levels. The following primer pairs were used:

hHPRT fwd. (5′- CCGGCTCCGTTATGGC -3′),

hHPRT rev. (5′- GGTCATAACCTGGTTCATCATCA -3′),

hephrinB2 fwd. (5′- AGTTCGACAACAAGTCCCTTTG -3′),

hephrinB2 rev. (5′- AGCAATCCCTGCAAATAAGG -3′),

hZEB2 fwd. (5′- AACAAGCCAATCCCAGGAG -3′),

hZEB2 rev. (5′- ACCGTCATCCTCAGCAATATG -3′),

hTWIST1 fwd. (5′- CTACGCCTTCTCGGTCTGG -3′),

hTWIST2 rev. (5′- CTCCTTCTCTGGAAACAATGAC -3′),

hTWIST2fwd. (5′- AGCAAGATCCAGACGCTCAAG -3′),

hTWIST2 rev. (5′- GGAGAAGGCGTAGCTGAGG -3′),

hGAPDH fwd. (5′- TGAACGGGAAGCTCACTGG -3′),

hGAPDH rev. (5′- TCCACCACCCTGTTGCTGTA -3′).

### siRNA transfection

To inhibit gene expression of HIF-1α and HIF-2α, siRNA were purchased from Eurogentec. siRNA transfections were performed twice at 24 h intervals with Oligofectamine (Invitrogen). To repress gene expression of the EMT repressors, LN229 cells were transiently transfected with a 20 nM pool of three ON-TARGETplus siRNA oligonucleotides (Thermo Scientific) as above. Cells were transfected 2 days prior to mRNA and protein level analysis, as described above.

### Luciferase reporter assay

The 1.7 kb DNA fragment upstream of the translation start of the murine *ephrinB2* gene was cloned into the pGL3 luciferase reporter vector. To generate the mutant ephrinB2–luciferase reporter (Supplementary Fig. 5) the three putative ZEB2-binding sites (CACCTN) were altered to GGTACC using a site directed mutagenesis PCR with the KAPA Hifi DNA polymerase (Peqlab Biotechnologie GmbH) according to the manufacturer's protocol. G55TL cells were transfected with the wild-type or mutant pGL3 ephrinB2–luciferase constructs (Supplementary Fig. 7) and an SV40-Renilla luciferase expressing construct (Promega, Madison, USA) serving as an internal control. Cells were grown for 48 h under adherent or sphere conditions±18 h under 1% O_2_ condition as described and assayed for luciferase activity with the Dual-Luciferase Reporter Assay System (Promega, Madison/USA). ZEB2 expression was achieved by transfection with the pCS3-SIP1FL plasmid kindly provided by G. Berx (Gent, Belgium).

### Chromatin immunoprecipitation

The EZ-Magna chromatin immunoprecipitation A kit (Millipore) was used according to the manufacturer's instructions. 3–4 × 10^7^ LN229 cells were lysed in 1.5 ml lysis buffer. Lysates were sonified on ice with a Branson SONIFIER 250 equipped with a 3 mm double stepped micro tip, 10 times for 10 s with a 30 s break at a constant output level of 4. The immunoprecipitation was performed with 500 μl lysate, 45 μl protease inhibitors, 405 μl dilution buffer, 50 μl beads and 10 μg of rabbit anti-ZEB2 antibody (Sigma, SAB2102760) or rabbit IgG as a negative control for 20 h at 4 °C. The quantitative RT–PCR was done as described above using primers specific for the ZEB2-binding site at positions 107188327–107188332 on chromosome 13 in the human ephrinB2 promoter (which is homologous to the ZEB2 c site in the mouse ephrinB2 promoter—see Supplementary Fig. 7):

5′- GTGGAAACAGCGACGGCCGAGTAG -3′

5′- ATCTGCGGAGAAGGGACGCCGAG -3′.

### Human VEGF ELISA

Supernatants conditioned for 48 h, of which 24 h under hypoxic conditions (1% O_2_), were collected from 80% confluent cell cultures. Analysis of human VEGF in cell culture supernatants was performed using the DuoSet ELISA Development System according to the manufacturer's instructions (R&D Systems).

### Statistical analysis

Results are presented as mean+or±s.e.m. For comparisons between two groups, statistical analysis was performed using a two-tailed unpaired Student's *t*-test. For comparisons between multiple groups, one-way analysis of variance with a Bonferroni post-test was performed using GraphPad Prism. Statistical significance was defined as *P<0.05, **P<0.01 and ***P<0.001. Sample sizes were determined based on previous experience with analogous experiments. The experiments were not randomized and not blinded. For *in vivo* experiments, animals that unexpectedly died were excluded from the analysis.

### Data availability

The authors declare that all data supporting the findings of this study are available within the article and its Supplementary Information files.

## Additional information

**How to cite this article:** Depner, C. *et al*. EphrinB2 repression through ZEB2 mediates tumour invasion and anti-angiogenic resistance. *Nat. Commun.* 7:12329 doi: 10.1038/ncomms12329 (2016).

## Supplementary Material

Supplementary Information Supplementary Figures 1-10 and Supplementary References.
