# Supplementary material for: EphrinB2 repression through ZEB2 mediates tumour invasion and anti-angiogenic resistance
Source: Nat Commun. 2016 Jul 29;7:12329. doi: 10.1038/ncomms12329 (PMC4974575; doi:10.1038/ncomms12329)
Supplement: Supplementary Information — Supplementary Figures 1-10 and Supplementary References. [file ncomms12329-s1.pdf]

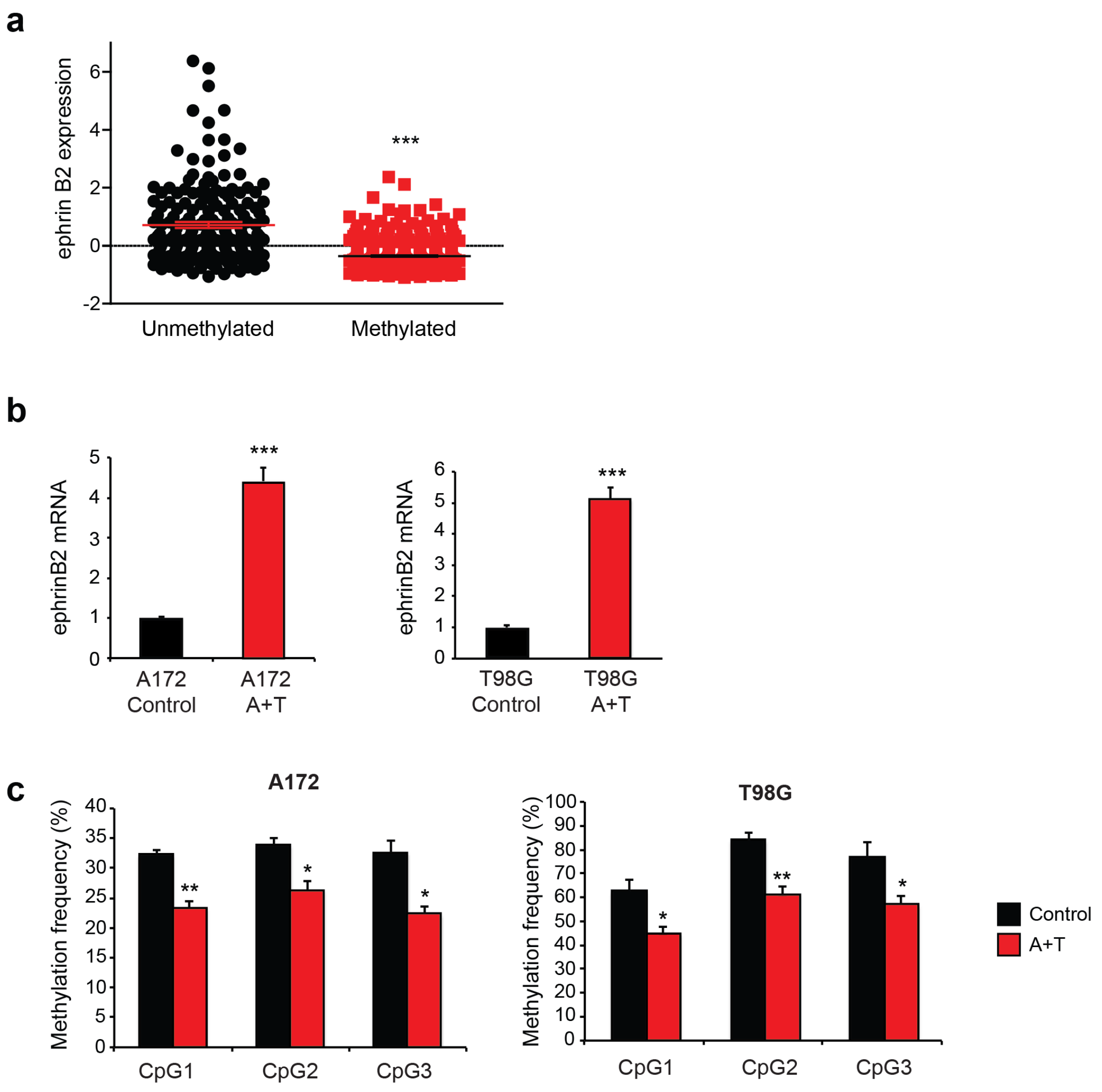


**Supplementary Figure 1. DNA demethylation increases ephrinB2 expression.**

**a,** EphrinB2 expression in gliomas of the TCGA cohort with an unmethylated and with a methylated ephrinB2 promoter (n=530 gliomas). **b,** qPCR analysis of ephrinB2 expression in A172 and T98G glioblastoma cells with hypermethylated ephrinB2 promoter, with or without treatment with 5-aza-2‘-deoxycytidin (500 nM) and trichostatin A (1 µM) for 48 h and 24 h, respectively (A+T) (n=3). **c,** Mean methylation frequency determined by pyrosequencing of sodium bisulfite-modified DNA (n=3). Note that the methylation frequency of the three investigated CpG dinucleotides located

at positions 107,186,901 (CpG1), 107,186,907 (CpG2) and 107,186,919 (CpG3) in the EFNB2 CpG-island (107,186,469 - 107,189,024 (http://genome.ucsc.edu)) decreases significantly after treatment with the demethylating agent 5-Aza-2‘-Deoxycytidin and Trichostatin A (A+T). Data are means + or ± SEM. *$P<0.05$, **$P<0.01$, ***$P<0.001$ using Student’s t-test.

a

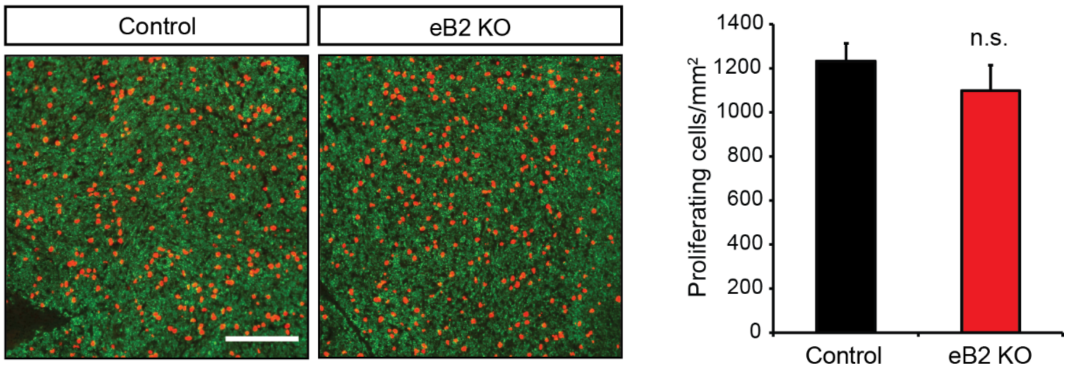


b

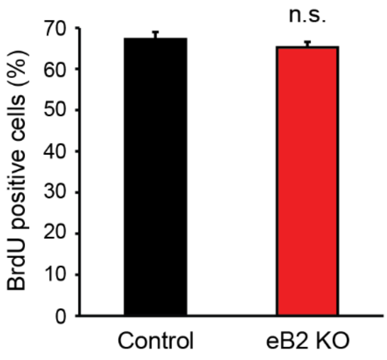


**Supplementary Figure 2. EphrinB2 deletion does not affect tumour proliferation**

**a,** Quantification of tumour cell proliferation as assessed by co-staining for phospho-histone H3 (P-H3) and human nuclei (hN) in control and ephrinB2 KO glioma xenografts (n=5-6 tumors). **b,** Quantification of tumour cell proliferation based on BrdU incorporation in cultured control and ephrinB2 KO glioma cells (n=6). Data are means + SEM. n.s., not significant using Student's t-test. Scale bar, 100 µm.

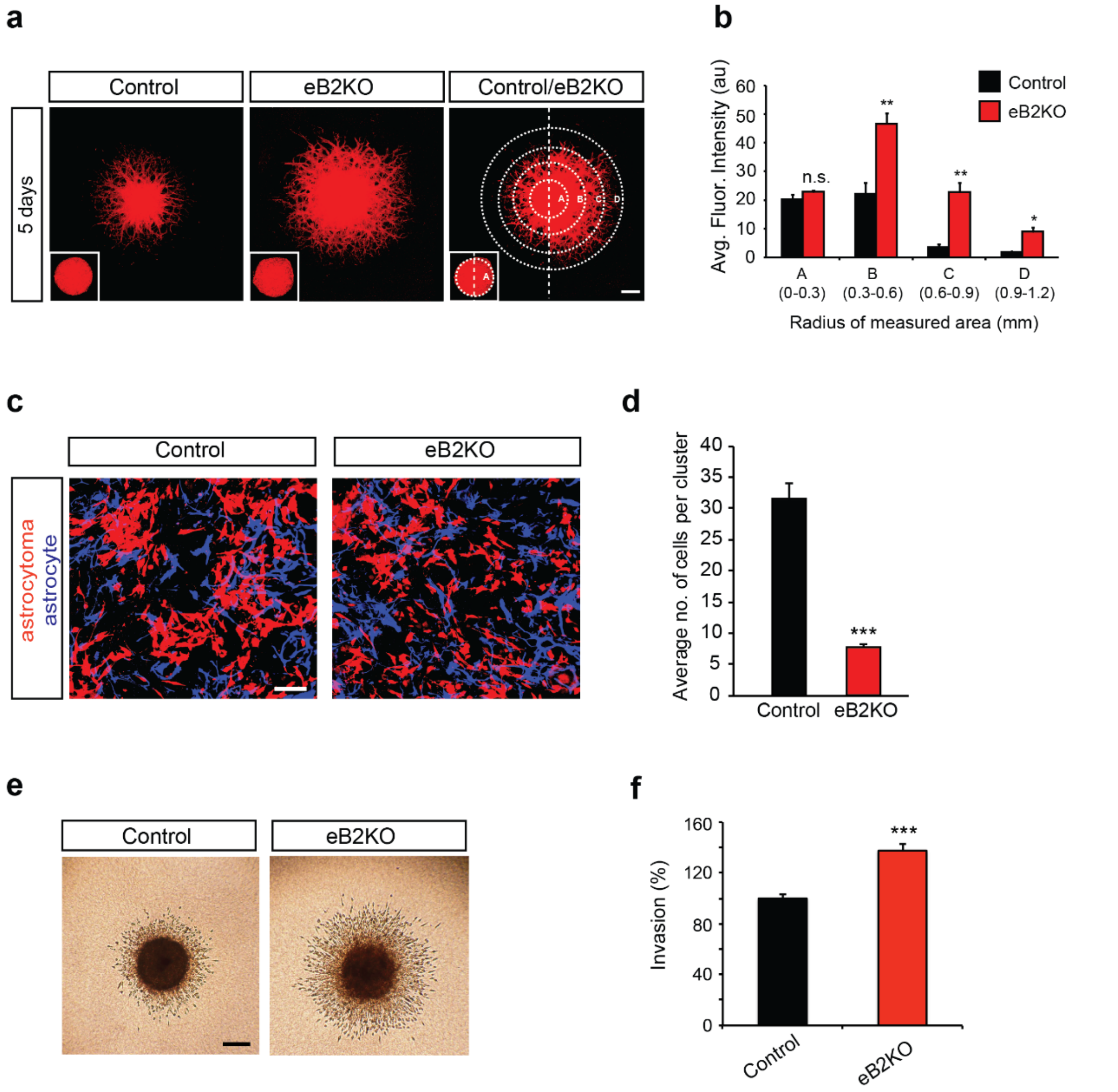


**Supplementary Figure 3. Loss of ephrinB2 increases glioma invasion.**

**a, b,** Glioma cells lacking ephrinB2 show increased invasion of brain slices. *Ex vivo* assay to investigate glioma invasion in organotypic brain slices. Invasion was analysed 5 days after spheroid seeding. Control and eB2KO spheroids on the day of seeding are shown in the insets (**a**). Quantification representing the fluorescence intensity in concentric areas from the spheroid centre; A-D correspond to radial strips with radii increments of 0.3 mm each (n=8 spheroids) (**b**). **c**,**d**, EphrinB2 KO glioma cells demonstrate increased dispersal and intermingling with co-cultured astrocytes. Representative confocal images of wild-type (control) or ephrinB2 KO (eB2KO)

glioma cells (red) co-cultured with wild-type astrocyte cells (blue). Images were taken after 48 h of co-culture (**c**). The number of glioma cells per cell cluster from 4-5 random optical fields per condition were counted (n=5 experiments). (**d**). **e,f,** Invasion of ephrinB2 KO glioma spheroids in a collagen gel is significantly increased. Invasion was quantified by measuring the percentage of invaded area normalized to the spheroid rim (n=30 spheroids). Data are means + SEM. Scale bars, 300μm (**a**), 100 μm (**c, e**). *$P<0.05$, **$P<0.01$, *** $P<0.001$ using Student's t-test.

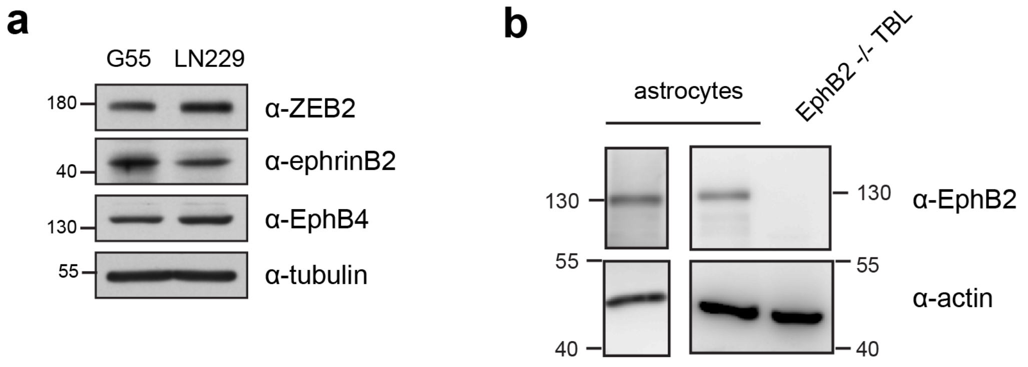


**Supplementary Figure 4. Expression levels of ZEB2, ephrinB2 and the ephrinB2 receptors EphB2 and EphB4**

**a,** Western blot for ZEB2, ephrinB2 and EphB4 in G55 and LN229 glioblastoma cells.

**b,** Western blot for EphB2 and EphB4 in astrocytes. Total brain lysates (TBL) of EphB2 knockout mice were used as a control. Actin was used as a loading control.

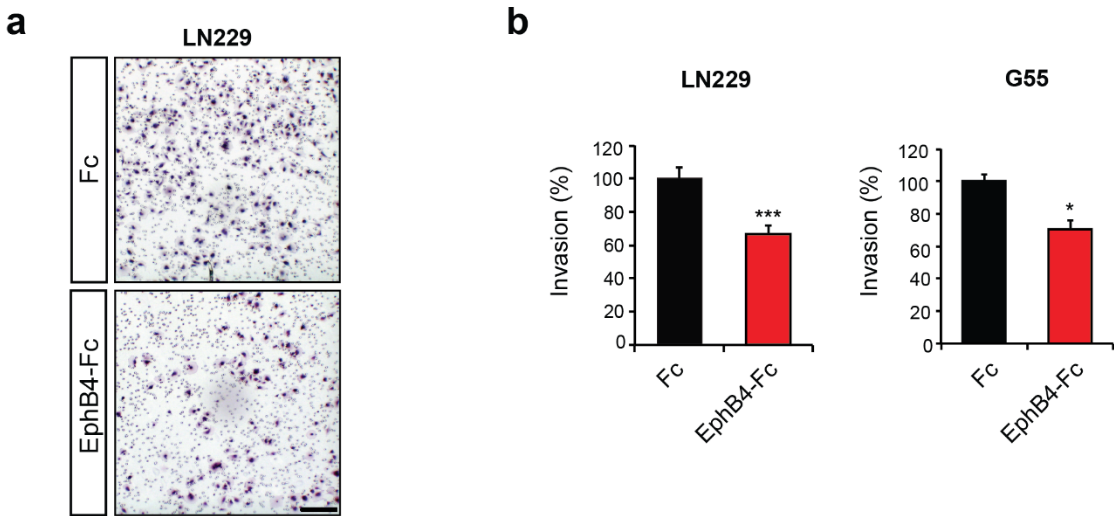


**Supplementary Figure 5. EphrinB2 activation inhibits glioma invasion in vitro.** **a,b,** LN229 and G55 glioma cell invasion was quantified in a Boyden chamber assay using filters coated with Matrigel containing pre-coupled EphB4-Fc to stimulate ephrinB2 signaling or Fc control. Representative images of HE stained LN229 cells showing reduced numbers of invaded tumour cells at the bottom side of filters coated with EphB4-Fc containing Matrigel (**a**). Quantification of the area covered by invaded cells normalized to Fc control (n=12) (**b**). Data are means + SEM. Scale bar, 200 μm *$P$<0.05, *** $P$<0.001 using Student's t-test.

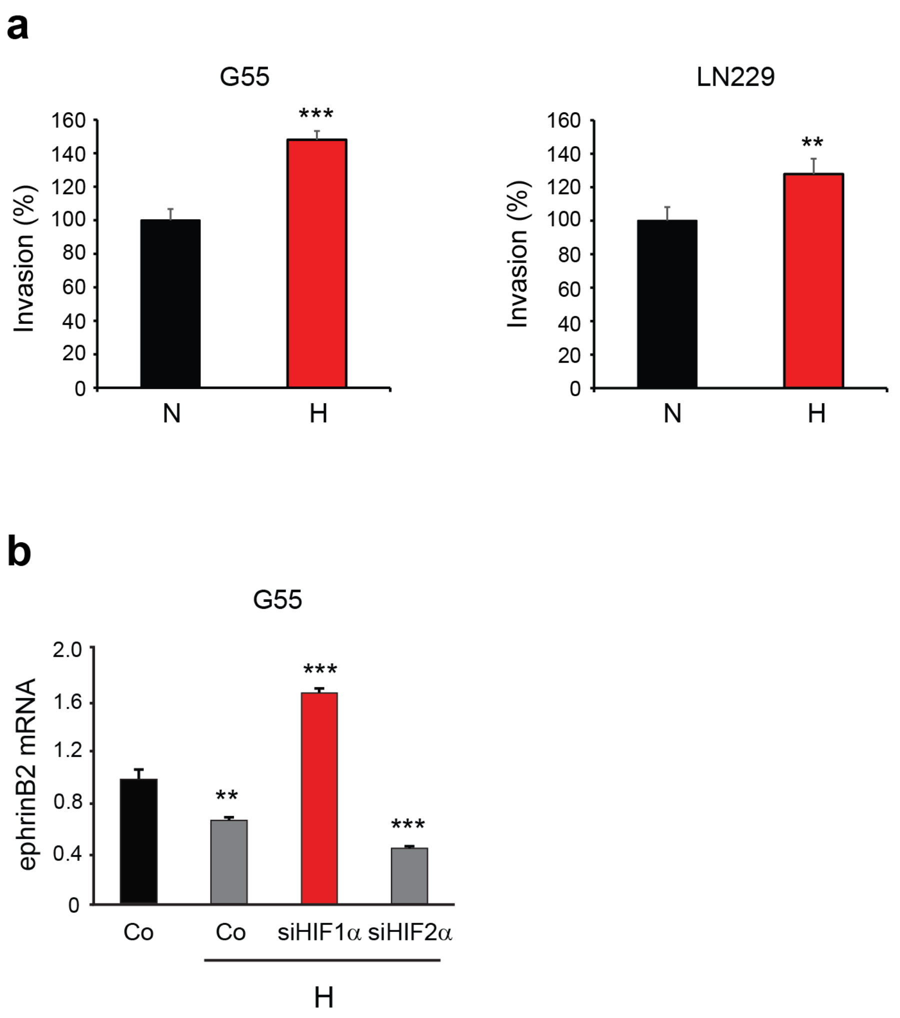


**Supplementary Figure 6. Hypoxia induces glioma cell invasion and downregulates ephrinB2 expression through HIF-1α.**

**a,** Hypoxia promotes glioma cell invasion. Invasion of G55 and LN229 glioblastoma cells cultured under 21%$O_2$ (N) or 1%$O_2$ (H) for 24h was assessed using a modified Boyden chamber assay (n=6). **b,** qRT-PCR analysis of ephrinB2 mRNA levels in G55 glioblastoma cells expressing scrambled control, HIF-1α or HIF-2α shRNA following exposure to 1% $O_2$ (H) for 18 h (n=3). Data are means + SEM. **P*<0.05, ***P*<0.01, ****P*<0.001 using Student's t-test (**a**) or one-way ANOVA with Bonferroni post-test (**b**, ANOVA *P*<0.0001).

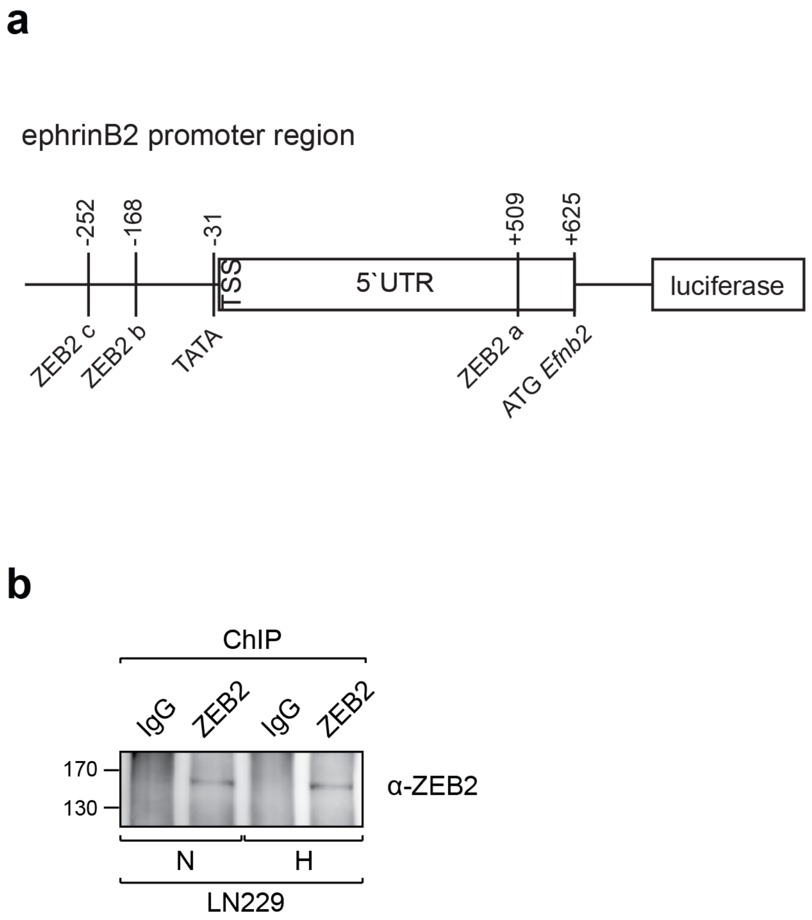


**Supplementary Figure 7. EphrinB2 reporter construct and ZEB2 ChIP.**

**a,** Schematic representation of the regulatory sequence of the murine ephrinB2 gene [1] used in the luciferase wild type and mutant reporter constructs (see Fig. 4b); the location of the 5'-UTR is indicated, as previously described [1]. Zeb a, Zeb b and Zeb c indicate the positions of the binding sites for ZEB2 that are conserved between mouse and human and that were altered in the mutant reporter construct. **b,** Western blot for ZEB2 in LN229 cells, following chromatin immunoprecipitation (ChIP) with an anti-ZEB2 antibody or control IgG under 21% $O_2$ (N) or 1% $O_2$, (H).

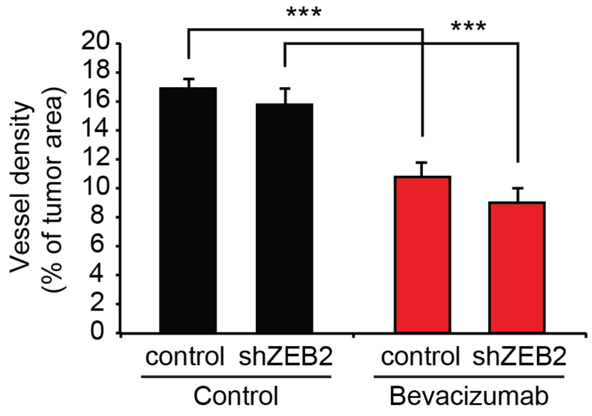


**Supplementary Figure 8. Bevacizumab treatment, but not ZEB2 inhibition reduces blood vessel density.**

Quantification of the density of blood vessels, immunostained with anti-podocalyxin antibodies in intracranial tumour xenografts of GFP expressing polyclonal G55 pools, stably transduced with scrambled control or ZEB2 shRNA following IgG control or bevacizumab treatment (n=8 tumours). Data are means + SEM. ***$P$<0.001 using one-way ANOVA with Bonferroni post-test (ANOVA $P$<0.0001).

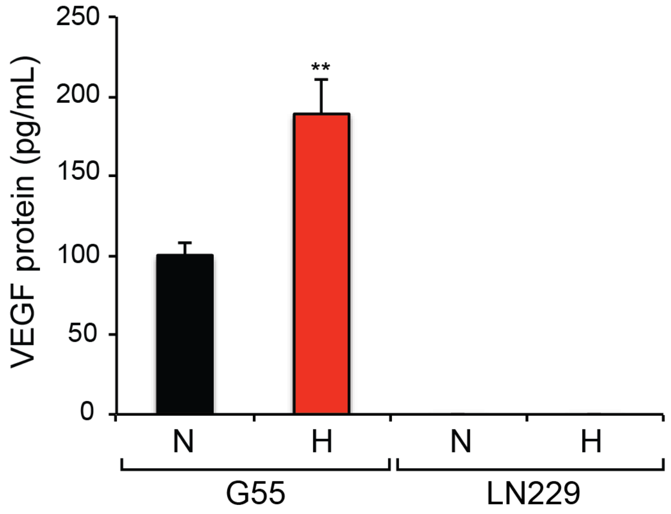


**Supplementary Figure 9. LN229 glioblastomas do not produce VEGF.**

Production of VEGF is induced by hypoxia in G55 glioblastoma cells. No VEGF is detected in LN229 conditioned cell culture medium. Quantification of secreted VEGF protein in conditioned culture medium from G55 and LN299 glioblastoma cells grown at 21% $O_2$ (N) or 1% $O_2$ (H) for 18h by ELISA (n=4). Data are means + SEM. **$P$<0.01 using Student's t-test.

Fig. 2a

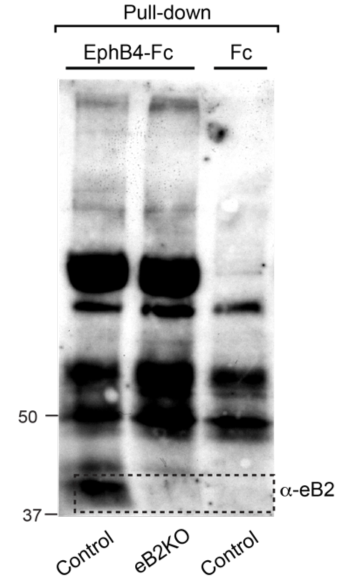


Fig. 2g

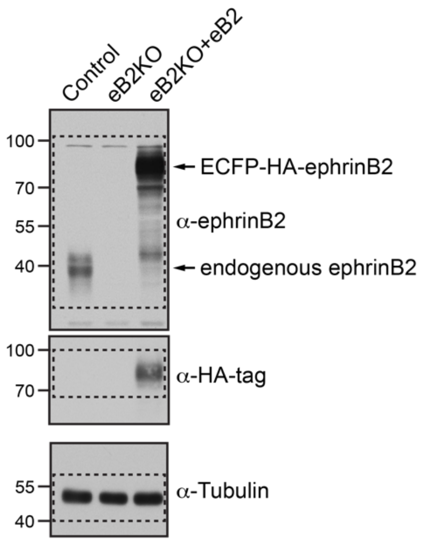


Fig. 3f

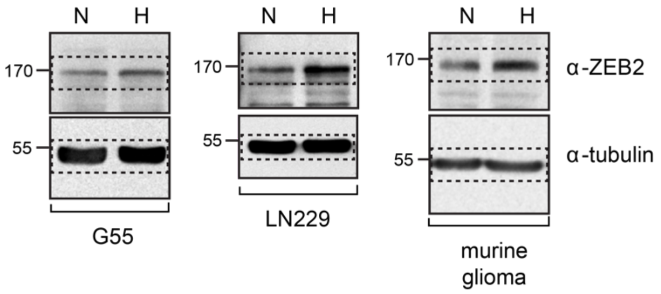


Fig. 3g

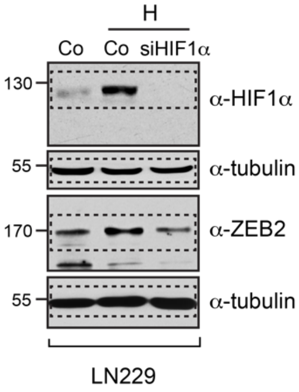


Fig. 3h

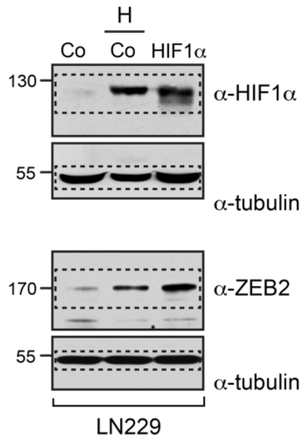


Fig. 4c

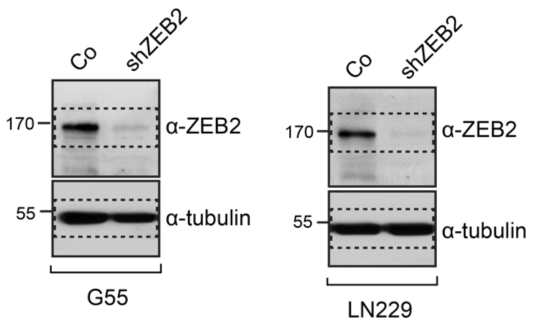


**Supplementary Figure 10. Uncropped, full-size scans of Western Blots**

Uncropped, full-size scans of Western Blots shown in Fig. 2a, 2g, 3f-h, 4c.

Fig. 4e

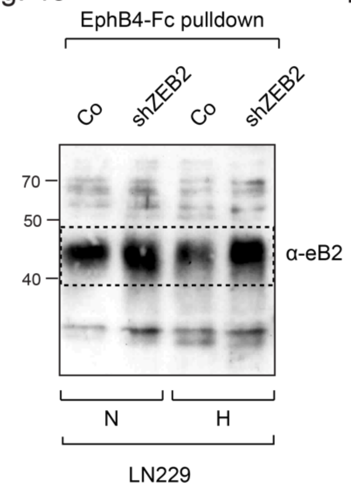


Fig. 7d

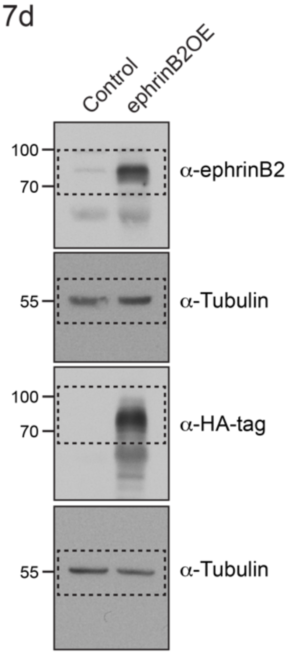


Suppl. Fig. 4a

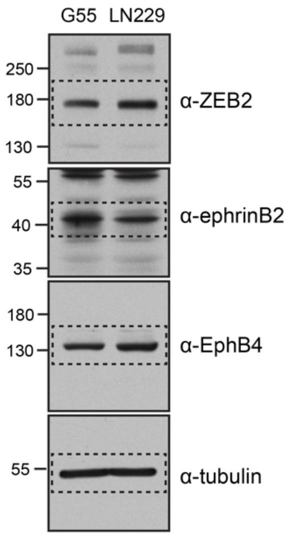


Suppl. Fig. 4b

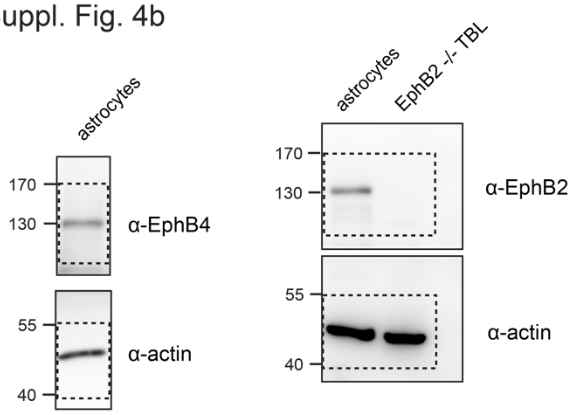


Suppl. Fig. 7b

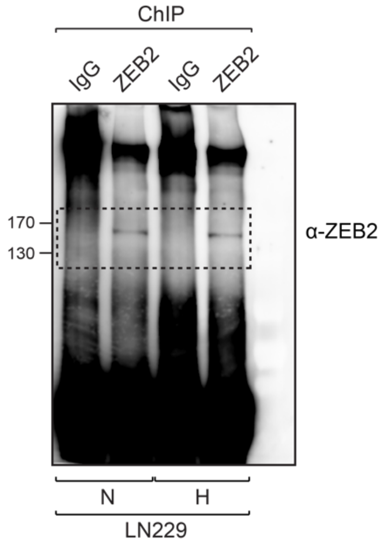


**Supplementary Figure 10 cont. Uncropped, full-size scans of Western Blots**

Uncropped, full-size scans of Western Blots shown in 4e, 7d, Supplementary Fig. 4a,b and Supplementary Fig. 7b

**Supplementary References**

1. Sohl M., Lanner F., Farnebo F. Characterization of the murine Ephrin-B2 promoter. *Gene* **437**, 54-59 (2009).
